# Racial discrimination and adverse pregnancy outcomes: a systematic review and meta-analysis

**DOI:** 10.1136/bmjgh-2022-009227

**Published:** 2022-06-27

**Authors:** Kim Robin van Daalen, Jeenan Kaiser, Samuel Kebede, Gabriela Cipriano, Hassan Maimouni, Ekiomoado Olumese, Anthea Chui, Isla Kuhn, Clare Oliver-Williams

**Affiliations:** 1 Cardiovascular Epidemiology Unit, Department of Public Health and Primary Care, Cambridge University, Cambridge, UK; 2 Faculty of Medicine & Dentistry, University of Alberta, Edmonton, Alberta, Canada; 3 Department of Medicine, Albert Einstein College of Medicine, Bronx, New York City, USA; 4 Cayetano Heredia University, Lima, Peru; 5 University of East Anglia, Norwich, UK; 6 Vanderbilt University School of Medicine, Nashville, Tennessee, USA; 7 School of Clinical Medicine, University of Cambridge, Cambridge, UK; 8 Medical Library, School of Clinical Medicine, University of Cambridge, Cambridge, UK

**Keywords:** maternal health, public health, systematic review, child health

## Abstract

**Introduction:**

Racial discrimination has been consistently linked to various health outcomes and health disparities, including studies associating racial discrimination with patterns of racial disparities in adverse pregnancy outcomes. To expand our knowledge, this systematic review and meta-analysis assesses all available evidence on the association between self-reported racial discrimination and adverse pregnancy outcomes.

**Methods:**

Eight electronic databases were searched without language or time restrictions, through January 2022. Data were extracted using a pre-piloted extraction tool. Quality assessment was conducted using the Newcastle–Ottawa Scale (NOS), and across all included studies using the Grading of Recommendations Assessment, Development and Evaluation (GRADE) approach. Random effects meta-analyses were performed on preterm birth and small for gestational age. Heterogenicity was assessed using Cochran’s χ^2^ test and I^2^ statistic.

**Results:**

Of 13 597 retrieved records, 24 articles were included. Studies included cohort, case–control and cross-sectional designs and were predominantly conducted in the USA (n=20). Across all outcomes, significant positive associations (between experiencing racial discrimination and an adverse pregnancy event) and non-significant associations (trending towards positive) were reported, with no studies reporting significant negative associations. The overall pooled odds ratio (OR) for preterm birth was 1.40 (95% CI 1.17 to 1.68; 13 studies) and for small for gestational age it was 1.23 (95% CI 0.76 to 1.99; 3 studies). When excluding low-quality studies, the preterm birth OR attenuated to 1.31 (95% CI 1.08 to 1.59; 10 studies). Similar results were obtained across sensitivity and subgroup analyses, indicating a significant positive association.

**Conclusion:**

These results suggest that racial discrimination has adverse impacts on pregnancy outcomes. This is supported by the broader literature on racial discrimination as a risk factor for adverse health outcomes. To further explore this association and underlying mechanisms, including mediating and moderating factors, higher quality evidence from large ethnographically diverse cohorts is needed.

**What is already known on this topic**

- When assessing subjective (self-reported) and objective health outcomes in cross-sectional and longitudinal studies, consistent associations have been found between racial discrimination and poor health outcomes.
- Similarly, racial disparities in pregnancy outcomes may be rooted in social factors, such as perceived racial discrimination.
- However, thus far, limited research has assessed the total evidence on the impact of racial discrimination on adverse pregnancy outcomes.

**What this study adds**

- As racial and/or ethnic disparities worsen or persist in fetal, neonatal and maternal health outcomes, with racialised people experiencing worse outcomes, it is pertinent to identify and address the underlying causal and mediating factors of these disparities, beyond traditional biomedical risk factors.
- This systematic review and meta-analysis expands our understanding of the mechanisms by which racism creates health disparities by examining the available peer-reviewed evidence base on the impact of perceived or self-reported racial discrimination on adverse pregnancy outcomes, including low birth weight, very low birth weight, small for gestational age, preterm birth and hypertensive disorder of pregnancy.

**How this study might affect research, practice or policy**

- In accordance with evidence on other health outcomes, our review highlights that racial discrimination has adverse impacts on pregnancy outcomes.
- To further explore this association and its underlying mechanisms, including mediating and moderating factors, higher quality evidence on fetal, neonatal and maternal health outcomes using a life course approach and large ethnographically diverse cohorts is needed.

## Introduction

James Marion Sims, a controversial 19th-century figure often credited as the ‘Father of Gynaecology,’ developed revolutionary tools and surgical techniques used in modern obstetrics and gynaecology, most notably the Sims’ speculum. However, he was also a known racist, conducting experiments needed for these developments on unconsenting enslaved black women without anaesthesia,^1 2^ placing these experiments among the likes of the Tuskegee syphilis experiment^3^ and Henrietta Lacks^4^ in a long history of unethical experimentation and exploitation of racial minorities. Today, instruments continue to bear his name in routine obstetrics and gynaecology practice,^1 2^ with more broadly embedded racism continuing to translate to worse pregnancy outcomes among racialised communities.

Maternal mortality rates among black and indigenous women in the USA are 2–3 times higher than in white women.^5^ Similarly, in the UK, maternal mortality rates are 2–4 times higher among black and Asian women compared with white women.^6^ For several decades, race has been recognised in the literature and medical/public health curricula as a social determinant of health and a risk factor for numerous diseases.^7–10^ During COVID-19, societal inequalities were brought to the forefront by exacerbating existing health inequities and injustices disproportionately affecting racialised populations.^11–14^ Historical attempts to explain racial and/or ethnic disparities in health have explored differential expressions of genetic and biological factors. However, health disparities between population groups cannot simply be explained by biological factors alone.^15^ Evidence increasingly suggests that upstream social, environmental, economic and political factors are fundamental drivers of health inequities, and that it is often not race, but racism, that is largely the root cause of racialised health disparities. A recent study examined the relationship between self-identified race and socially assigned race with general health status. The results indicated that among Hispanic, indigenous and mixed-race individuals, those who were perceived by others as being white experienced significantly better health than those perceived as being non-white. As health disparities persisted between seemingly white and non-white racialised individuals despite belonging to a shared racial background, this suggests that social factors such as racial discrimination may play an important role in determining health outcomes.^16^

Race is a socially constructed category that impacts health through race-associated differences in individuals’ material conditions, access to resources, experiences, opportunities and interactions within society. Racism is a system of structuring opportunity and assigning value based on the social interpretation of an individual’s perceived ‘race’ that disadvantages some individuals and communities while advantaging others. A growing body of epidemiological evidence documents the health impacts of racism.^17^ A meta-analysis of 293 studies reported that racism was associated with poorer general, physical and mental health, without being moderated by age, sex, birthplace or education level.^18^ Other studies have found similar results.^19–21^

In particular, disparities in fetal, neonatal and maternal health outcomes have been reported, with racialised women experiencing worse outcomes. While racialised socioeconomic disparities can be linked to upstream structural racism, stark disparities persist between non-white and white individuals of a similar socioeconomic background. An American integrative review in 2015 reported that the majority of studies found a relationship between racial discrimination and pregnancy outcomes, even after accounting for socioeconomic status.^22^ Moreover, although it has been demonstrated that black women with higher educational attainment have better outcomes than black women with lower educational attainment, they continue to have worse outcomes than white women with lower educational attainment.^23^ Thus, the impacts of institutional and interpersonal (ie, personally mediated) racism may more directly relate to disparities in pregnancy outcomes.^24–26^

While studies suggest that existing patterns of racial disparities in pregnancy outcomes are rooted in social factors like perceived racial discrimination, the cumulative peer-reviewed evidence base on racial discrimination has, thus far, not been comprehensively synthesised and assessed. Therefore, to expand our understanding of the mechanisms, this systematic review and meta-analysis assesses the association between perceived racial discrimination and adverse pregnancy outcomes.

## Methods

### Search strategy and selection criteria

The study protocol was registered prospectively with PROSPERO (https://www.crd.york.ac.uk/prospero/) (CRD42020224691). Findings were reported following the Preferred Reporting Items for Systematic Reviews and Meta-Analyses (PRIMSA) guidelines (online supplemental table 4).^27^ For this systematic review, we searched eight electronic databases (PubMed, Medline, EMBASE, Scopus, CINAHL, Web of Science, PsycINFO, and SocINDEX), without language or lower bound year restrictions, to 19 November 2020. An updated search was conducted on 11 January 2022. Using a controlled vocabulary, we applied search terms related to ‘pregnant women’, ‘racial discrimination’, and ‘adverse pregnancy outcomes’, informed by previous reviews^18 28 29^ and a medical information specialist (IK). The full search strategy is provided in online supplemental table 1. We cross-referenced bibliographies of relevant publications (eg, reviews, reports) and the included studies in the full-text screening to identify any additional eligible studies.

10.1136/bmjgh-2022-009227.supp1 Supplementary data

For this study, we focused on self-reported/perceived racial discrimination. Following previous studies,^30^ perceived discrimination is defined as discrimination perceived or experienced by members of a certain group. This includes unjust behaviour, attitude, judgement or treatment experienced by a racial group.^31^ Events that the law deems ‘not discriminatory’ can still be perceived as discriminatory, therefore these were also included in our definition of perceived discrimination.^30 32^ Likewise, events deemed discriminatory by law may have been excluded if they were not experienced as discriminatory by the individual.^30 32^ Perceived discrimination can take various forms, including personal or institutional level, conscious or unconscious, and subtle or direct.^33^

We included both neonatal and maternal adverse pregnancy outcomes, including preterm birth (PTB), low birth weight (LBW) and hypertensive disorder of pregnancy (HDP). A full list is given in online supplemental table 2.

### Study selection

Studies that met the inclusion criteria were peer-reviewed quantitative studies which (1) reported on the association between self-reported discrimination and adverse pregnancy outcomes, (2) included a measure of perceived/self-reported racial discrimination, race prejudice or racism as an exposure, (3) reported at least one adverse pregnancy outcome, and (4) were conducted on pregnant women or women who were previously pregnant. Studies were excluded if they were (1) non-human studies, conference proceedings, reviews, (2) lacking a full text, or (3) on biological males or girls under 16 years of age.

Studies were selected in two stages. After removing duplicates, abstracts and titles were double-screened using the selection criteria by eight researchers using the software Rayyan (https://rayyan.ai/). In the second stage, full texts of studies that met the selection criteria were retrieved and double-screened by eight researchers. Any disagreements between researchers were discussed among two authors until consensus was reached. Non-English papers were translated or reviewed by a native/fluent speaker of the research team.

### Data extraction

Data from included studies were independently extracted by five researchers using a pre-piloted extraction tool. The following information was extracted for each study: author, year, study title, study design, study population and characteristics (eg, age, country), recruitment procedures used, total number of participants, number of controls, definition and ascertainment of racial discrimination (eg, experience of discrimination scale, racism and lifetime experience scale), number of individuals categorised as experiencing discrimination, adverse pregnancy outcome(s) reported, ascertainment of outcome(s), percentage/N of individuals with outcome, association measures, adjusted variables and an open field for additional information. When relevant information could not be obtained from four articles, authors were contacted.

### Study quality assessment

Three researchers independently assessed the quality of individual studies using the Newcastle–Ottawa Scale (NOS) to assess risk of bias.^34^ The final score was converted to Agency for Healthcare Research and Quality (AHRQ) standards of good, fair and poor (see online supplemental figures 1–3). To assess the risk of bias across all included studies, the Grading of Recommendations Assessment, Development and Evaluation (GRADE) approach was applied.^35^ Evidence from observational studies starts at low quality due to residual confounding and bias, among other issues. When serious study limitations were identified, the evidence was downgraded by one level. These limitations included imprecision in effect estimates, serious inconsistency, risk of bias, potential publication bias and indirectness of evidence. Any disagreements between researchers were discussed among two authors until consensus was reached. Due to the limited and heterogenous evidence base, no studies were excluded from the research synthesis based on their assessed quality.

### Meta-analysis

To be included in the meta-analysis, studies had to report an estimated measure of association (eg, odds ratio (OR), hazard ratio (HR), relative risk (RR), prevalence ratio (PR), correlation or β coefficients), or sufficient information to calculate a measure. One researcher explored whether articles reported sufficient data to be included in meta-analyses, and all excluded articles were discussed with a second researcher. At least three studies needed to report on the same outcome in order to be included. A narrative synthesis by adverse pregnancy outcome was conducted for studies excluded from the meta-analysis.

The most commonly used metric for measuring association was OR and 95% CI in the papers reviewed, and was employed as the measure of effect size in the meta-analyses. As PRs and HRs are not necessarily interchangeable with ORs, other measures of association were converted to ORs, or unadjusted ORs were calculated based on the studies’ available data. Unadjusted ORs were computed with the available information for the studies by Braveman *et al*,^36^ Slaugther-Acey *et al*,^37^ Misra *et al* 38 and Fryer *et al*.^39^ When both unadjusted and adjusted ORs were available, we pooled the adjusted ORs. We took this approach even when ORs were adjusted for different variables, as it is more likely that adjusted effect estimates are representative of the true effect than crude ORs.

Meta-analyses for PTB and small for gestational age (SGA) were conducted with STATA Version 16 and 17 (StataCorp, Texas, USA) using the meta-set, meta-forestplot, meta-funnelplot and meta-bias commands.^40^ Due to the anticipated heterogeneity between studies, a Der Simonian and Laird random effects meta-analysis was performed. To quantify heterogeneity we used Cochran’s χ^2^ test and generated an I^2^ statistic as a percentage of variability. I^2^ values of 75%, 50% and 25% correspond to high, moderate and low heterogeneity.^41^ Two-tailed p values <0.05 were considered statistically significant, except where otherwise specified.

We conducted several sensitivity analyses. The meta-forestplot leaveoneout command was used to explore the influence of individual studies on the pooled effect size.^40^ Further sensitivity analyses were performed on (1) crude ORs of included studies (for those that had crude ORs available or which could be calculated), (2) fair and good quality studies, (3) subgroups of participant race or ethnicity, and (4) reported adjusted HRs, PRs and ORs as approximates of each other (instead of the computed crude ORs). Under the rare disease assumption, OR and RR may be used as approximates of each other^42^ and HR may be considered as an extension for uncommon outcomes.^43 44^

The influence of publication bias was assessed using the Begg’s and Egger’s test (p<0.10 representing statistical significance),^45^ and graphically using (contour-enhanced) funnel plots. This was done for outcomes with a minimum of 10 unique included studies.^46^ We used a non-parametric trim-and-fill method to estimate the number of studies potentially missing due to publication bias and to provide bias-adjusted results. This method is based on the assumption that there should be a symmetrical funnel plot.^47 48^

### Patient involvement

No patients were involved in the conceptualisation or conduct of this study due to the nature of the study as a systematic review.

## Results

### Characteristics of the included studies

**Figure 1:**
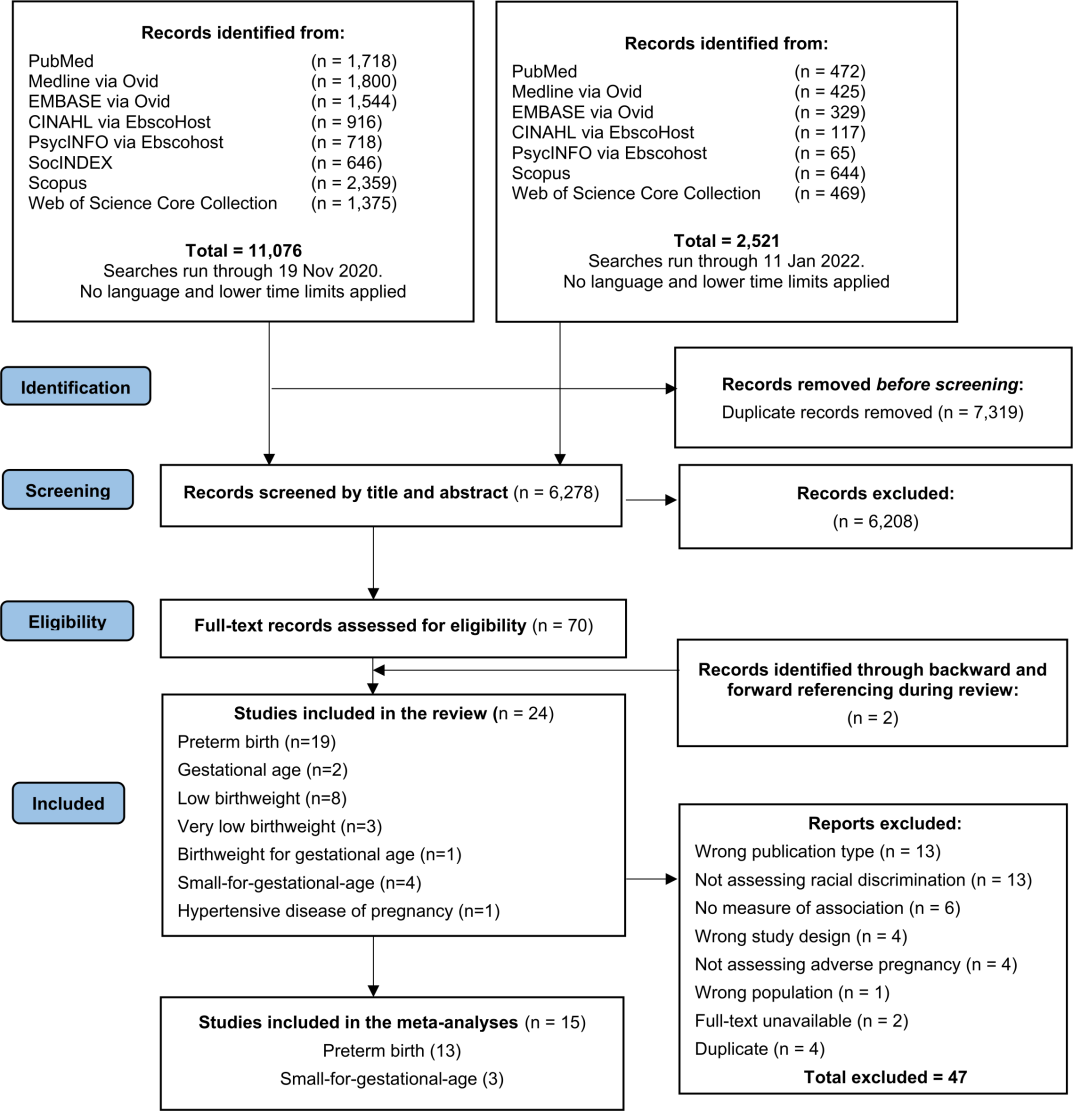
PRISMA flow diagram of search strategy.^27^

A total of 11 076 publications were retrieved from the databases in the first run and 2521 in the second run (figure 1). After removing duplicates, 6278 studies were screened by title and abstract and 70 by full text. Twenty-four articles were included in this review with summary characteristics reported in table 1 and results in table 2.^36–39 49–68^ Most studies were conducted in the USA (n=20),^36–39 49 50 52 54–58 60–63 65–69^ and four studies were conducted in other countries: Germany (n=1),^51^ Australia (n=1),^59^ New Zealand (n=1),^53^ Serbia (n=1)^64^ and Macedonia (n=1).^64^ Included studies had a cohort design (n=14),^37–39 49–59^ case–control design (n=4)^60–63^ or cross-sectional design (n=6).^36 64–68^ The number of included participants ranged from 39^70^ to 9470.^58^ Although no time restrictions were applied, all studies were published after 1999. Study periods ranged from 1992^49^ to 2016.^51^

**Table 1:** Summary characteristics of included documents

Study	Study design	Country	Population	Pregnancy type	Race or ethnicity included	Ascertainment racial discrimination	Outcome	Ascertainment outcome
Barber and Robinson^68^	Cross-sectional study	USA	No of participants: 2634 Age range: 15–44 Women part of the Virginia Pregnancy Risk Assessment Monitoring System (PRAMS) recruited from 47 states, New York City, Puerto Rico and the District of Columbia	NR	Non-Hispanic black, non-Hispanic white, Hispanic, non-Hispanic other	Answer to a self-reported question on the PRAMS questionnaire	PTB (<37 weeks’ gestation) LBW (<2500 g) SGA (based on 10th percentile)	Birth certificate data
Brown *et al* 2019^59^	Cohort study	Australia	No of participants: 344 (165 reporting discrimination or unfair treatment) Mean age: 25 (range 15–43) Women were recruited in urban, regional and remote areas of South Australia over 2 years. Twelve Aboriginal research interviewers recruited women via public hospitals, community-based agencies, community events and their own community networks. To be eligible to take part, women needed to have given birth to an Aboriginal infant in South Australia between June 2011 and July 2013, and to be aged ≥14 years at the time of the birth	NR	Aboriginal	Adapted questions of Indigenous Racism Experience questionnaire (adapted from Paradies *et al*)^103^	LBW (<2500 g), PTB (birth <37 weeks gestation), SGA	Did not report; Australian birth weight standards
Dixon *et al* 2012^55^	Cohort study	USA	No of participants: 539 mother–infant pairs (176 reporting 1–2 domains of racism, 187 ≥3) Mean age: 30 (5.8) Subjects were participants in Project Viva, a prospective cohort study of gestational factors, pregnancy outcomes and offspring health. Between 1999 and 2002 women who attended their initial prenatal visit at eight obstetric offices of a multi-specialty group practice in eastern Massachusetts were recruited	Singleton	Black, Hispanic, Asian, other	Adapted or expanded Experiences of Discrimination scale measuring lifetime racial discrimination (from Krieger *et al*, 1990,^104^ 2005)^105^	Birth weight for gestational age	US national reference data; length and height measurements performed by research assistants following standardised techniques
Dominguez *et al* 2010^67^	Cross-sectional study	USA	No of participants: 124 Mean age: 28.65 (5.14) African American, 31.43 (4.07) non-Hispanic white Approached by a research nurse in the prenatal clinic of a large urban medical centre in Los Angeles County or referred by private practitioners with admitting privileges to this medical centre. To be eligible, potential participants had to be ≥18 years, fluent in English, and at no more than 18 weeks gestation with a singleton pregnancy	Singleton	African-American, non-Hispanic white	Answer to a self-reported question on perceived racism (loosely based on items from Krieger *et al*)^104^	Birth weight (g), gestational age at delivery, LBW, preterm delivery	Medical records
Janevic *et al* 2017^64^	Cross-sectional study	Serbia, Macedonia	No of participants: 410 women (top 25% on EDS were experiencing discrimination) Age: <20: 73; 20–24: 168; 25–29: 106; ≥30: 63 Romani women in Belgrade, Serbia and Skopje, North Macedonia in 2012–2013 who had given birth in the previous 2 years	NR	Romani	Everyday Discrimination Scale (developed by Williams *et al*)^106^	LBW (<2500 g)	Self-report
Mustillo *et al* 2004^49^	Cohort study	USA	No of participants: 352 (171 experiencing racism) Mean age: 33.1 (3.3) black women, 34.8 (3.2) white women From original 1985 prospective cohort study CARDIA, recruited from four geographically diverse metropolitan areas. Participants who gave birth between 1992 and 1995 were eligible	Singleton	Black, white	Self-reported experiences of racial discrimination based on Krieger’s Experiences of Discrimination scale (from Krieger *et al*)^104^	LBW (<2500 g), preterm deliveries (<37 weeks gestation)	Self-report (at year 10)
Thayer *et al* 2019^53^	Cohort study	New Zealand	No of participants: 1653 (ever verbal attack 427, ever physical attack 59, unfair treatment 498) Mean age: 29.5 (5.6) Sample from Growing Up in New Zealand (GUINZ) study recruiting pregnant women who had an estimated birth date between 25 April 2009 and 25 March 2010 and living within geographic area defined by Auckland, Counties-Manukau or Waikato District Health Board regions in the North Island of Aotearoea, New Zealand.	Singleton	Māori, Pacific and Asian	Questionnaire asking about lifetime and past experience of unfair treatment (Growing Up in New Zealand questionnaire)^107^	LBW (<2500 g), short gestation length (37 weeks gestation)	Medical records
Collins *et al* 2000^62^	Case–control study	USA	No of participants: 85 (25 cases, 60 controls) Age: no overall statistic clearly reported Subjects were recruited from Children’s Memorial Hospital (neonatal intensive care unit) and Cook County Hospital (neonatal intensive unit, normal newborn nursery) in Chicago	Singleton	African-American	Questionnaire on perception and exposure to racial discrimination during pregnancy (from Krieger *et al*)^104^	VLBW (<1500 g)	Medical records
Collins *et al* 2004^63^	Case–control study	USA	No of participants: 312 (104 cases, 208 controls) Age: no overall statistic clearly reported Mothers delivering at Cook County Hospital and University of Chicago Hospital in Chicago between 1 November 1997 and 31 October 2000. Cases: mothers of singleton births with VLBW infants and preterm. Controls: mothers of critically ill, singleton non-LBW term infants admitted to neonatal ICU or healthy singleton non-LBW infants admitted to newborn nursery	Singleton	African-American	Questionnaire on perception and exposure to racial discrimination during pregnancy (adapted from Krieger *et al* 104 and McNeilly *et al*)^108^	VLBW (<1500 g), PTB (<37 weeks gestation)	Medical records
Lespinasse *et al* 2004^60^	Case–control study	USA	No of participants: 312 women (141 reporting racial discrimination) Age: <19: 82; 19–29: 156; ≥30: 64 Cases were 104 mothers of VLBW infants recruited from the admission logbooks of the NICUs of Cook County Hospital and the University of Chicago Hospital. Controls were 104 mothers of NBW; healthy infants (‘healthy controls’); and 104 mothers of NBW, sick infants who required assisted ventilation or other major life support (‘sick controls’). Healthy controls were recruited from the labour and delivery logbooks and were chosen if admitted near the same date as the cases; sick controls were identified from NICU logbook	Singleton	African-American	Questionnaire that included questions on racism	VLBW (<1500 g)	Medical records
Braveman *et al* 2017^36^	Cross-sectional study	USA	No of participants: 10 323 women (812 black women (36.9%) reporting chronic worry from discrimination, 446 white women (5.5%)) Age: >15 California Maternal and Infant Health Assessment (MIHA) Cross-sectional California statewide representative surveys of 2201 black and 8122 white, non-Latino, US-born postpartum women with singleton live births during 2011–2014. The MIHA sample is drawn randomly from statewide birth certificate data each year	Singleton	Black, white non-Latino	Questionnaire asking about chronic worry for race-based unfair treatment	PTB (17–36 weeks of gestation)	Birth certificate information and National Centre for Health Statistics criteria; obstetric estimate or LMP for GA
Daniels *et al* 2020^65^	Cross-sectional study	USA	No of participants: 173 Age range: 30–50 US-born, post-partum women from African-AmericanWomen’s Heart & Health Study (AAWHHS), which includes detailed health information on a community sample of 208 African-American women aged 30–50 residing in the San Francisco Bay Area	NR	African-American	Adapted Everyday Discrimination Scale, adapted a scale (adopted from Krieger *et al*)^105^	Preterm labour (<37 weeks gestation)	Self-report
Dole *et al* 2003^56^	Cohort study	USA	No of participants: 1962 (574 reporting some or higher discrimination) Age range: 16–19: 274; 20–29: 1036; ≥30: 652 The Pregnancy, Infection, and Nutrition Study, a prospective cohort study of risk factors for PTB, recruited women from two prenatal clinics in central North Carolina who were between 24 and 29 weeks’ gestation, beginning in August 1995	Singleton	African-American	Psychological self-reported questionnaire (adapted from Krieger,^104^ Krieger and Sidney)^109^	PTB (<37 weeks gestation)	Medical records
Dole *et al* 2004^57^	Cohort study	USA	No of participants: 1898 (364 black women reporting discrimination, 271 white women) Age range: 16–19: 261; 20–29: 1002; ≥30: 635 The Pregnancy, Infection, and Nutrition (PIN) Study was conducted in central North Carolina at two prenatal care sites (>16 years of age, between 24 and 29 weeks gestation. Self-reported white or African American).	Singleton	African-American, white	Adapted Experience of Discrimination Scale (Krieger) (adapted from Krieger^104^ Krieger and Sidney)^109^	PTB (<37 weeks gestation)	Medical records; algorithm using LMP or earliest US before 20 weeks
Fryer *et al* 2020^39^	Cohort study	USA	No of participants: 1732 (736 reporting medium or high discrimination) Mean age: 24.2 (5.0) non-Hispanic African American, 25.7 (5.2) Latina Community Child Health Research Network Study, a 5-year, multisite, prospective cohort study. Women were recruited from Baltimore, Chicago, North Carolina, Los Angeles and Washington DC from 2008 to 2012 and followed up over a 2-year period. 18–40 years of age	Singleton	African-American, Latina	Everyday Discrimination Scale (developed by Williams *et al*)^106^	Spontaneous preterm delivery (<37 weeks)	Medical records
Giurgescu *et al* 2012^66^	Cross-sectional study	USA	No of participants: 72 (no reporting discrimination not reported) Mean age: 23.38 (5.44) full term, 23.27 (5.24) preterm African-American women ≥18 years old, at least 24 weeks gestation at birth, at least 24 hours after birth and medically stable in Chicago hospital. Excluded if had medical comorbidity, unstable or had medically indicated birth	Singleton	African-American	Adapted Experiences of Discrimination scale (adapted from Krieger *et al,* 1990,^104^ 2010^110^	PTB (<37 weeks gestation)	Medical records based on LMP and confirmed by US
Misra *et al* 2010^38^	Cohort study	USA	No of participants: 832 (425 experiencing racism) Mean age: 23.1 (5.6) African-American women residing in Baltimore, Maryland, enrolled prenatally if they received care at one of three Johns Hopkins Medical Institution (JHMI) prenatal clinics or enrolled post-partum if they delivered at JHMI with late, none or intermittent prenatal care	Singleton	Black or African-American, Hispanic	Racism and Lifetime Experiences Scale (RaLES), RaLES Daily Life Experiences Scale (DLE), Racism‐Related Experiences Scale (RRE) (from Harrell *et al*)^111^	PTB (<37 weeks gestation), gestational age	Medical records
Rankin *et al* 2011^61^	Case–control study	USA	No of participants: 277 (160 cases, 117 controls) Age: <20: 60; 20–34: 186; ≥35: 31 African-American mothers delivering at Stroger Hospital of Cook County and Unversity of Chicago Hospital in Chicago, IL between July 2001 and June 2005	Singleton	African-American	Adapted Perceived Racism Scale (adapted from McNeilly *et al*)^108^	PTB (<37 weeks gestation) LBW (<2500 g)	Medical records
Rosenberg *et al* 2002^50^	Cohort study	USA	No of participants: 4966 Age range: 21–69 Follow-up study from Black Women’s Health Study (1995) recruiting US African-American women. Those completing 1997–1999 questionnaires and reported singleton birth were eligible	Singleton	African-American	Nine questions regarding their experience of racial discrimination	PTB (<37 weeks gestation)	Self-report
Scholaske *et al* 2019^51^	Cohort study	Germany	No of participants: 2515 (46 experiencing racism) Mean age: 31.43 (5.44) German autochthonous, 30.71 (5.68) Turkish immigrant women Sample from German Socio-Economic Panel (SOEP) study of German autochthonous and Turkish immigrant women who recently gave birth, within first year after delivery	Singleton	German autochthonous Turkish	Self-report: single item asking frequency of perceived discrimination	PTB (<37 weeks gestation)	Self-report (from newborn questionnaire)
Slaughter-Acey *et al* 2016^37^	Cohort study	USA	No of participants: 1232 women (DLE-B score second quartile 305, third 307, fourth 315) Age range: 21–69 Sample from the Life-course Influences of Fetal Environments (LIFE) study which recruited black or African-American women aged 18–45 years who gave birth to a singleton at a surburban hospital in metropolitan Detroit	Singleton	Black or African-American	RaLES Daily Life Experiences Scale (DLE) scale of Racism and Bother (from Harrell *et al*)^111^	PTB (<37 weeks gestation)	Medical records; algorithm to estimate GA (based on early US, LMP, later US or clinician’s estimate)
Wheeler *et al* 2018^54^	Cohort study	USA	No of participants: 1606 women (1256 black, 350 white) Mean age: primiparous: 23.5 (5.8); multiparous term birth: 26.7 (6.2); multiparous PTB: 27.7 (5.6) English-speaking pregnant women enrolled in Healthy Pregnancy, Healthy Baby Study in Durham, North Carolina, USA. Participants received their care at the Duke Obstetrics Clinic of the Durham County Health Department Prenatal Care	Singleton	Non-Hispanic black, non-Hispanic white	Perceived Racism Scale (from Dole *et al*,^57^ adopted Krieger *et al* 104 and Krieger and Sidney)^109^	PTB (spontaneous)	Medical records; enrolment interview
Slaughter-Acey *et al* 2019^52^	Cohort study	USA	No of participants: 778 (730 acknowledged at least one item of racism, 48 did not acknowledge any item) Mean age: 23.3 (5.7) Black or African-American women living in Baltimore City, Maryland who attended JHMI satellite prenatal clinic or delivered a live infant at JHMI	NR	Black or African-American	Adapted Racism and lifetime Experience Scale (RaLES) (from Harrell *et al*)^111^	SGA	SGA determined using cut-off point for birth weight ratio continuous measure (observed birth weight divided by expected birth weight for infant’s GA)
Grobman *et al* 2018^58^	Cohort study	USA	No of participants: 9470 women (N reporting racial discrimination not reported) Age: no overall statistic clearly reported The Nulliparous Pregnancy Outcomes Study: Monitoring Mothers-To-Be is a prospective cohort study in which 10 038 nulliparous women (>13 years of age) with singleton pregnancies were enrolled from geographically diverse hospitals affiliated with eight clinical centres	Singleton	Non-Hispanic white, non-Hispanic black, Hispanic, Asian, other	Krieger Racism Scale (developed by Krieger *et al*)^105^	Hypertensive disorder of pregnancy, SGA, PTB (<37 weeks gestation)	Medical records

GA, gestational age; LBW, low birth weight; LMP, last menstrual period; NICU, newborn intensive care unit; NR, not reported; PTB, preterm birth; SGA, small for gestational age; US, ultrasound; VLBW, very low birth weight.

**Table 2:** Results of included studies

Study	Exposure and outcome	Adjustments		Results	
Barber and Robinson^68^	***Exposure*** Self-reported racial discrimination during the 12 months before the new baby was born			*All participants, OR (95%* *CI)*	
		Race/ethnicity /ethnicity, age, income, health insurance status		LBW: 2.27 (1.18 to 4.38)	
		Race/ethnicity, age, education		PTB: 1.76 (0.90 to 3.43)	
		Race/ethnicity, education, income, marital status		SGA: 1.31 (0.70 to 2.45)	
				*Non-Hispanic white, OR (95%* *CI)*	
		Age, education, income, health insurance status, marital status		LBW: 0.30 (0.08 to 1.14)	
	***Outcome*** LBW (<2500 g), PTB (<37 weeks gestation), SGA (based on 10th percentile)	Age, education, income, and marital status		PTB: 0.13 (0.03 to 0.61)	
		Age, education, income, health insurance status, marital status, smoking during pregnancy		SGA: 1.47 (0.42 to 5.13)	
				*Non-Hispanic black, OR (95% CI)*	
		Age, income, health insurance status		LBW: 3.56 (1.28 to 9.91)	
		Age, education, income, drinking during pregnancy, gestational diabetes		PTB: 7.18 (2.28 to 22.65)	
		Age, income, drinking during pregnancy		SGA: 1.95 (0.55 to 6.87)	
Brown *et al* 2019^59^	***Exposure*** Self-reported racism and discrimination in perinatal care			*OR (95% CI), crude*	
		NA		LBW: 2.1 (1.1 to 4.1)	
		NA		PTB: 1.0 (0.5 to 1.9)	
		NA		HBW: 0.8 (0.4 to 1.8)	
		NA		SGA: 2.0 (1.1 to 3.6)	
		NA		LGA: 1.0 (0.4 to 2.4)	
				*Model 1, OR (95% CI)*	
		Parity		LBW: 2.1 (1.1 to 4.1)	
		Parity		HBW: 0.8 (0.4 to 1.8)	
		Parity		PTB: 1.0 (0.5 to 1.9)	
		Parity		SGA: 2.0 (1.1 to 3.6)	
		Parity		LGA: 1.0 (0.4 to 2.4)	
	***Outcome*** LBW (<2500 g), PTB (birth <37 weeks gestation), SGA			*Model 2, OR (95%* *CI)*	
		Parity, use of cigarettes and/or cannabis during pregnancy		LBW: 1.9 (1.0 to 3.8)	
		Parity, use of cigarettes and/or cannabis during pregnancy		HBW: 0.9 (0.4 to 1.9)	
		Parity, use of cigarettes and/or cannabis during pregnancy		PTB: 1.0 (0.5 to 1.9)	
		Parity, use of cigarettes and/or cannabis during pregnancy		SGA: 1.9 (1.0 to 3.5)	
		Parity, use of cigarettes and/or cannabis during pregnancy		LGA: 1.0 (0.4 to 2.5)	
				*Model 3, OR (95%* *CI)*	
		Parity, stressful events and stressful events/social health issues		LBW: 2.0 (1.0 to 3.9)	
		Parity, stressful events and stressful events/social health issues		HIB: 0.8 (0.3 to 1.7)	
		Parity, stressful events and stressful events/social health issues		PTB: 1.1 (0.5 to 2.1)	
		Parity, stressful events and stressful events/social health issues		SGA: 1.7 (0.9 to 3.2)	
		Parity, stressful events and stressful events/social health issues		LGA: 0.9 (0.4 to 2.2)	
Dixon *et al* 2012^55^	***Exposure*** Self-reported racism during their lifetime			*Birth weight for gestational age z-score, overall, Model 1*	
		Maternal age, race/ethnicity, child sex, age at outcome		1–2 racism domains: −0.04 (−0.24 to 0.15)	
				3+ racism domains: −0.18 (−0.38 to 0.01)	
				*Birth weight for gestational age z-score, overall, Model 2*	
		Maternal age, race/ethnicity, child sex, age at outcome, maternal nativity, education, pregnancy BMI, household income		1–2 racism domains: −0.11 (−0.31 to −0.09)	
				3+ racism domains: NA	
				*Birth weight for gestational age z-score, black, Model 1*	
		Maternal age, race/ethnicity, child sex, age at outcome		1–2 racism domains: 0.00 (−0.29 to 0.28)	
				3+ racism domains: 0.00 (−0.27 to 0.27)	
				*Birth weight for gestational age z-score, black, Model 2*	
		Maternal age, race/ethnicity, child sex, age at outcome, maternal nativity, education, pregnancy BMI, household income		1–2 racism domains: −0.01 (−0.31 to 0.28)	
				3+ racism domains: −0.05 (−0.34 to 0.23)	
	***Outcome*** Birth weight for gestational age			*Birth weight for gestational age z-score, Hispanic, Model 1*	
		Maternal age, race/ethnicity, child sex, age at outcome		1–2 racism domains: −0.04 (−0.41 to 0.34)	
				3+ racism domains: −0.52 (−0.95 to −0.10)	
				*Birth weight for gestational age z-score, Hispanic, Model 2*	
		Maternal age, race/ethnicity, child sex, age at outcome, maternal nativity, education, pregnancy BMI, household income		1–2 racism domains: −0.25 (−0.63 to 0.14)	
				3+ racism domains: −0.70 (−1.13 to −0.26)	
				*Birth weight for gestational age z-score, Asian, Model 1*	
		Maternal age, race/ethnicity, child sex, age at outcome		1–2 racism domains: −0.07 (−0.46 to 0.33)	
				3+ racism domains: −0.18 (−0.66 to 0.30)	
				*Birth weight for gestational age z-score, Asian, Model 2*	
		Maternal age, race/ethnicity, child sex, age at outcome, maternal nativity, education, pregnancy BMI, household income		1–2 racism domains: −0.11 (−0.51 to 0.30)	
				3+ racism domains: −0.21 (−0.70 to 0.27)	
Dominguez *et al* 2010^67^	***Exposure*** Self-reported lifetime and childhood-vicarious racism	Gestational age, medical risk, spontaneous labour, clinic patient, parent’s education		*Hierarchical linear regression models racism as predictors of birth weight*	
				*Lifetime (β) gestational age, African-American*	
				−0.28	
	***Outcome*** LBW <2500 g, birth weight (g), gestational age at delivery, PTB (birth <37 weeks gestation)			*Childhood-vicarious (β), African-American*	
				−0.01	
				*Lifetime (β) gestational age, non-Hispanic white*	
				−0.26*	
				*Childhood-vicarious (β), non-Hispanic white*	
				−0.07	
Janevic *et al* 2017^64^	***Exposure*** Self-reported interpersonal racism during daily life ***Outcome*** LBW <2500 g			*LBW, RR (95%* *CI)*	
		NA		Crude: 2.1 (1.0 to 4.2)	
		Age, parity, years at current residence, household wealth index		Model 1: 2.4 (1.1 to 5.2)	
		Age, parity, years at current residence, household wealth index, institutional discrimination measures		Model 2: 2.5 (1.0 to 5.9)	
Mustillo *et al* 2004^49^	***Exposure*** Self-reported racial discrimination during their lifetime ***Outcome*** LBW (<2500 g), PTB (birth <37 weeks gestation)			*LBW, OR (95% CI), Model 6*	
		Race/ethnicity		1–2 racism domains: 2.04 (0.50 to 8.31)	
				3+ racism domains: 4.81 (1.50 to 15.40)	
				*LBW, OR (95% CI), Model 8*	
		Race/ethnicity, smoking status, alcohol use, depressive symptomatology, education, income, pregnancy net wain gain		1–2 racism domains: 1.96 (0.51 to 7.56)	
				3+ racism domains: 4.98 (1.43 to 17.39)	
				*LBW, OR (95% CI), Model 9*	
		Race/ethnicity, smoking status, alcohol use, depressive symptomatology, education, income, pregnancy net wain gain, gestational age		1–2 racism domains: 1.06 (0.29 to 3.84)	
				3+ racism domains: 1.56 (0.32 to 7.76)	
				*PTB, OR (95% CI), Model 2*	
		Race/ethnicity		1–2 racism domains: 1.97 (0.89 to 4.38)	
				3+ racism domains: 2.42 (1.03 to 5.69)	
				*PTB, OR (95% CI), Model 4*	
		Race/ethnicity, smoking status, alcohol use, depressive symptomatology, education, income		1–2 racism domains: 2.05 (0.93 to 4.50)	
				3+ racism domains: 3.05 (1.29 to 7.24)	
Rankin *et al* 2011^61^	***Exposure*** Self-reported racial discrimination during lifetime and past year ***Outcome*** LBW (<2500 g), PTB (birth <37 weeks gestation)	NA		*LBW-PTB, OR (95% CI), crude* High lifetime: 1.5 (0.9 to 2.8) High past-year: 2.5 (1.2 to 5.2)	
Thayer *et al* 2019^53^	***Exposure*** Self-reported racial discrimination across the lifetime and past year. ***Outcome*** Birth weight, gestational age		*Birth weight, β coeff (95% CI), Māori*	*Birth weight, β coeff (95% CI), Pacific*	*Birth weight, β coeff (95% CI), Asian*
		Maternal age, education, relationship status, smoking, BMI, offspring sex, household income	Any personal attack: −84.7 (−190 to 20.9)	Any personal attack: 24.8 (−109 to 159)	Any personal attack: 75.7 (−16.1 to 167)
			UT health: −13.0 (−166 to 140)	UT health: 142 (−46.7 to 330)	UT health: 4.98 (−194 to 204)
			UT work: −243 (−425 to −60.2)	UT work: 137 (−46.7 to 321)	UT work: 49.2 (−66.0 to 164)
			UT housing: −146 (−286 to −5.93)	UT housing: 15.1 (−177 to 207)	UT housing: 188 (7.04 to 369)
			UT criminal system: −95.8 (−255 to 64.0)	UT criminal system: 138 (−91.6 to 369)	UT criminal system: 2.12 (−295 to 299)
			UT banking: −122 (−333 to 89.7)	UT banking: 246 (−34.4 to 527)	UT banking: 119 (−314 to 553)
			UT education: −63.9 (−193 to 65.7)	UT education: 79.9 (−108 to 268)	UT education: −86.6 (−239 to 66.3)
			*Gestational length, β coeff (95% CI), Māori*	*Gestational length, β coeff (95% CI), Pacific*	*Gestational length, β coeff (95% CI), Asian*
		Maternal age, education, relationship status, smoking, BMI, offspring sex, household income	Any personal attack: −0.18 (−0.53 to 0.16)	Any personal attack: 0.29 (−0.12 to 0.70)	Any personal attack: 0.13 (−0.15 to 0.42)
			UT health: −0.36 (−0.87 to 0.15)	UT health: 0.25 (−0.32 to 0.84)	UT health: −0.26 (−0.88 to 0.36)
			UT work: −0.95 (−1.56 to −0.34)	UT work: −0.40 (−0.60 to 0.52)	UT work: 0.18 (−0.17 to 0.55)
			UT housing: −0.20 (−0.67 to 0.26)	UT housing: 0.09 (−0.50 to 0.69)	UT housing: 0.30 (−0.26 to 0.87)
			UT criminal system: −0.55 (−1.08 to −0.02)	UT criminal system: 0.51 (−0.19 to 1.22)	UT criminal system: 0.45 (−0.47 to 1.39)
			UT banking: −0.73 (−1.43 to −0.02)	UT banking: 0.64 (−0.21 to 1.51)	UT banking: 0.33 (−1.02 to 1.70)
			UT education: −0.24 (−0.67 to 0.18)	UT education: 0.18 (−0.39 to 0.76)	UT education: −0.31 (−0.79 to 0.16)
Collins *et al* 2000^62^	***Exposure*** Self-reported racial discrimination during pregnancy ***Outcome*** VLBW (<1500 g)			*VLBW, OR (95% CI)*	
		NA		Crude: 1.9 (0.5 to 6.6)	
		Maternal age, parity, social support		Model 1: 2.1 (0.7 to 6.1)	
		Smoking, alcohol, drug use		Model 2: 5.2 (1.2 to 22.0)	
		Maternal age, parity, social support, smoking, alcohol, drug use		Model 3: 3.3 (0.9 to 11.3)	
Collins *et al* 2004^63^	***Exposure*** Self-reported racial discrimination during pregnancy and lifetime ***Outcome*** VLBW (<1500 g), PTB (birth <37 weeks gestation)			*VLBW PTB, OR (95% CI), lifetime discrimination, crude*	
		NA		≥1 domain: 1.9 (1.2 to 3.1)	
				≥2 domains: 2.1 (1.2 to 3.8)	
				≥3 domains: 3.2 (1.5 to 6.6)	
				*VLBW PTB, OR (95% CI), discrimination during this pregnancy, crude*	
		NA		≥1 domain: 0.9 (0.5 to 1.7)	
				≥2 domains: 1.5 (0.5 to 4.4)	
				≥3 domains: NA	
				*VLBW PTB, OR (95% CI), lifetime discrimination*	
		Maternal age, education, cigarette smoking		≥1 domain: 1.7 (1.0 to 9.2)	
				≥3 domains: 2.6 (1.2 to 5.3)	
Lespinasse *et al* 2004^60^	***Exposure*** Self-reported racial discrimination ***Outcome*** VLBW (<1500 g)			*VLBW PTB, OR (95% CI)*	
		NA		1+ domain: 1.9 (1.2 to 3.0)	
				3+ domains: 2.7 (1.3 to 5.4)	
Braveman *et al* 2017^36^	***Exposure*** Self-reported chronic worry about racial discrimination			*PTB, PR (95% CI), US-born black*	
		NA		Crude: 1.73 (1.12 to 2.67)	
		Maternal age, parity, marital status, family income, maternal education, % of census-tract residents with income below poverty, number of major stressors during pregnancy, depressive symptoms during pregnancy		Model 1: 1.95 (1.27 to 2.97)	
		Maternal age, parity, marital status, family income, maternal education, % of census-tract residents with income below poverty, number of major stressors during pregnancy, depressive symptoms during pregnancy, smoked 3 months before pregnancy, binge drank while pregnant, unintended pregnancy, lack first-trimester prenatal care, interpregnancy interval, self-reported health pregnancy, diabetes, hypertension, underweight diagnoses before pregnancy, inadequate pregnancy weight gain		Model 2: 2.00 (1.33 to 3.01)	
				PTB, OR (95% CI), US-born black, crude*	
		NA		1.45 (1.1 to 1.9)*	
	***Outcome*** PTB (17–36 weeks gestation)			*PTB, PR (95% CI), US-born white*	
		NA		Crude: 1.77 95% CI (0.83 to 3.77)	
		Maternal age, parity, marital status, family income, maternal education, % of census-tract residents with income below poverty, number of major stressors during pregnancy, depressive symptoms during pregnancy		Model 1: 1.67 (0.73 to 3.79)	
		Maternal age, parity, marital status, family income, maternal education, % of census-tract residents with income below poverty, number of major stressors during pregnancy, depressive symptoms during pregnancy, smoked 3 months before pregnancy, binge drank while pregnant, unintended pregnancy, lack first-trimester prenatal care, interpregnancy interval, self-reported health pregnancy, diabetes, hypertension		Model 2: 1.84 (0.91 to 3.71)	
Daniels *et al* 2020^65^	***Exposure*** Self-reported direct and vicarious racial discrimination across three life stages (adult, adolescent, childhood)			*PTB, OR (95% CI), crude*	
		NA		Adult direct: 1.056 (0.888 to 1.257)	
				Adult vicarious: 1.140 (0.893 to 1.455)	
				Adolescent direct: 1.488 (1.006 to 2.202)	
				Adolescent vicarious: 1.265 (0.982 to 1.628)	
				Childhood direct: 1.086 (0.800 to 1.473)	
				Childhood vicarious: 1.449 (0.999 to 2.102)	
	***Outcome*** PTB (birth <37 weeks gestation)			*PTB, OR (95%* *CI)*	
		Number of pregnancies, income adjusted for household size, college educated, employed, marital status		Adult direct: 1.091 (0.914 to 1.302)	
				Adult vicarious: 1.131 (0.883 to 1.448)	
				Adolescent direct: 1.480 (1.002 to 2.187)	
				Adolescent vicarious: 1.271 (0.986 to 1.637)	
				Childhood direct: 1.100 (0.808 to 1.498)	
				Childhood vicarious: 1.453 (1.010 to 2.092)	
Dole *et al* 2003^56^	***Exposure*** Self-reported racial discrimination			*PTB, RR (95% CI), crude*	
		NA		Some discrimination: 0.8 (0.6 to 1.2)	
				Higher discrimination: 1.4 (1.0 to 1.9)	
	***Outcome*** PTB (birth <37 weeks gestation)			*PTB, RR (95%* *CI)*	
		Parity, poverty index		Some discrimination: 0.9 (0.6 to 1.4)	
				Higher discrimination: 1.4 (1.0 to 2.0)	
Dole *et al* 2004^57^	***Exposure*** Self-reported racial discrimination ***Outcome*** Preterm birth (birth <37 weeks gestation)			*PTB, RR (95% CI), African-American*	
		BMI, height		Some discrimination: 1.1 (0.6 to 2.1)	
				High discrimination: 1.8 (1.1 to 2.9)	
				*PTB, RR (95% CI), white*	
				Some discrimination: 0.8 (0.4 to 1.4)	
				High discrimination: NA	
Fryer *et al* 2020^39^	***Exposure***			*PTB, HR (95% CI), African-American, crude*	
		NA		Low: 0.8 (0.4 to 1.7)	
				Medium: 1.5 (0.8 to 2.9)	
				High: 1.4 (0.7 to 2.7)	
				*PTB HR 95% CI, African-American*	
		Geographic location		Low: 0.9 (0.4 to 1.9)	
				Medium: 1.6 (0.8 to 3.2)	
				High: 1.5 (0.7 to 3.1)	
	***Outcome*** PTB (spontaneous birth <37 weeks gestation)			*PTB HR 95% CI, Latina, crude*	
		NA		Low: 4.1 (1.1 to 14.6)	
				Medium: 4.0 (1.1 to 15.2)	
				High: 3.8 (0.9 to 15.1)	
				*PTB, HR (95% CI), Latina*	
		Geographic location		Low: 3.6 (0.99 to 13.2)	
				Medium: 4.1 (1.1 to 15.5)	
				High: 3.6 (0.9 to 14.4)	
				*PTB, OR (95% CI), African-American, crude* *	
		NA		High: 1.40 (0.68 to 2.85)*	
				*PTB, OR (95% CI), Latina, crude* *	
		NA		High: 3.82 (0.94 to 15.64)*	
Giurgescu *et al* 2012^66^	***Exposure*** Self-reported racial discrimination over the lifetime ***Outcome*** PTB (birth <37 weeks gestation)	Objective physical disorder, objective social disorder, violent crime, perceived physical disorder, perceived social disorder, perceived crime, EOD situation, EOD frequency, psychological distress		*PTB, OR (95% CI), crude*	
				EOD situation: 0.810 (0.50 to 1.32)	
				EOD frequency: 1.105 (0.93 to 1.31)	
Misra *et al* 2010^38^	***Exposure*** Self-reported racism over lifetime ***Outcome*** PTB (birth <37 weeks gestation) Gestational age	NA		*PTB, HR (95%* *CI), crude*	
				Above median RALES: 1.00 (0.70 to 1.44)	
				Top quartile in RALES: 0.88 (0.58 to 1.35)	
				*PTB, OR (95%* *CI), crud*e*	
				Top quartile in RALES: 0.88 (0.58 to 1.35)*	
Rosenberg *et al* 2002^50^	***Exposure*** Self-reported racial discrimination in daily life ***Outcome*** PTB (birth <37 weeks gestation)			*PTB, OR (95% CI), crude*	
		NA		Unfair treatment at job: 1.3 (1.1 to 1.7)	
				Unfair treatment in housing: 1.0 (0.8 to 1.3)	
				Unfair treatment by police: 1.2 (0.9 to 1.5)	
				People acting afraid of them at least once a week: 1.4 (1.0 to 2.0)	
				*PTB, OR (95% CI)*	
		Age of mother at birth, parity, marital status, previous preterm birth, mother born three or more weeks early, smoked during the pregnancy, years of educational attainment		Unfair treatment at job: 1.3 (1.1 to 1.6)	
				Unfair treatment in housing: 1.0 (0.8 to 1.3)	
				Unfair treatment by police: 1.1 (0.9 to 1.4)	
				People acting afraid of them at least once a week: 1.4 (1.0 to 1.9)	
Scholaske *et al* 2019^51^	***Exposure*** Frequency of self-reported racial discrimination in last year or within the last 2 years ***Outcome*** PTB (birth <37 weeks gestation)			*PTB, OR (95% CI), crude*	
		NA		Seldom/often: 4.25 (1.68 to 11.56)	
				*PTB, OR (95% CI*)	
		Infant sex, maternal age, parity, years of education		Seldom/often: 5.76 (1.95 to 19.38)	
Slaughter-Acey *et al* 2016^37^	***Exposure*** Self-reported racial discrimination during the index pregnancy and up to 1 year before the pregnancy ***Outcome*** PTB (birth <37 weeks gestation)	NA		*PTB, PR 95% (CI), crude*	
				Second quartile: 1.67 (1.16 to 2.40)	
				Third quartile: 1.32 (0.90 to 1.94)	
				Fourth quartile: 1.19 (0.81 to 1.76)	
				*PTB, OR (95% CI), crude* *	
				Second quartile: 1.85 (1.20 to 2.85)*	
				Third quartile: 1.4 (0.89 to 2.18)*	
				Fourth quartile: 1.23 (0.78 to 1.93)*	
Wheeler *et al* 2018^54^	***Exposure*** Self-reported racism during lifetime and pregnancy ***Outcome*** PTB (spontaneous birth <37 weeks gestation)			*PTB, OR (95% CI)*	
		Age, race, medical illness, psychiatric illness, tobacco use		Entire cohort: 1.06 (0.90 to 1.25)	
				Primiparous: 1.29 (0.91 to 1.83)	
				Multiparous prior term birth: 1.01 (0.79 to 1.30)	
				Multiparous prior PTB: 1.05 (0.78 to 1.40)	
Slaughter-Acey *et al* 2019^52^	***Exposure*** Self-reported overall racism during lifetime			*SGA, OR (95% CI), crude*	
		NA		Overall: 0.95 (0.82 to 1.10)	
				Personal: 0.92 (0.74 to 1.13)	
				Group: 0.97 (0.73 to 1.30)	
				*SGA, OR (95% CI), ≥25* years	
		Maternal education, Medicaid status, essential money, parity, recruitment status		Overall: 0.92 (0.66 to 1.28	
				Personal: 0.96 (0.58 to 1.58)	
				Group: 0.83 (0.47 to 1.45)	
	***Outcome*** SGA (using the cut point defined for the BW ratio continuous measure)			*SGA, OR (95% CI), 19–24* years	
		Maternal education, Medicaid status, essential money, parity, recruitment status		Overall: 0.86 (0.69 to 1.06)	
				Personal: 0.86 (0.64 to 1.18)	
				Group: 0.72 (0.47 to 1.10)	
				*SGA, OR (95% CI), ≤18* years	
		Maternal education, Medicaid status, essential money, parity, recruitment status		Overall: 1.45 (1.02 to 2.08)	
				Personal: 1.33 (0.86 to 2.09)	
				Group: 2.84 (1.10 to 7.32)	
Grobman *et al* 2018^58^	***Exposure*** Self-reported racism ***Outcome*** PTB (birth <37 weeks gestation), HDP, SGA			*OR (95% CI), non-Hispanic black*	
		Maternal age, BMI, smoking, medical comorbidities		Any PTB: 1.31 (1.04 to 1.64)	
				iPTB: 1.39 (1.00 to 1.93)	
				sPTB: 1.21 (0.90 to 1.63)	
				HDP: 0.98 (0.81 to 1.20)	
				SGA: 2.07 (1.69 to 2.53)	
				*OR (95% CI), Hispanic*	
		Maternal age, BMI, smoking, medical comorbidities		Any PTB: 0.95 (0.76 to 1.20)	
				iPTB: 0.93 (0.65 to 1.33)	
				sPTB: 0.95 (0.71 to 1.27)	
				HDP: 0.71 (0.58 to 0.86)	
				SGA: 1.45 (1.19 to 1.77)	
				*OR (95% CI), Asian*	
		Maternal age, BMI, smoking, medical comorbidities		Any PTB: 0.87 (0.56 to 1.36)	
				iPTB: 0.80 (0.37 to 1.74)	
				sPTB: 0.91 (0.54 to 1.53)	
				HDP: 0.82 (0.56 to 1.20)	
				SGA: 2.08 (1.54 to 2.81)	
				*OR (95% CI), other*	
		Maternal age, BMI, smoking, medical comorbidities		Any PTB: 1.14 (0.82 to 1.59)	
				iPTB: 1.00 (0.59 to 1.68)	
				sPTB: 1.23 (0.81 to 1.85)	
				HDP: 0.85 (0.63 to 1.14)	
				SGA: 1.42 (1.05 to 1.93)	

*These unadjusted ORs were computed with the available information in the study.

BMI, body mass index; EOD, experience of discrimination; HBW, high birth weight; HDP, hypertensive disorder of pregnancy; LBW, low birth weight; LGA, large for gestational age; PR, prevalence ratio; PTB, preterm birth; RR, relative risk/risk ratio; SGA, small for gestational age; UT, unfair treatment; VLBW, very low birth weight.

### Quality assessment of individual studies and overall evidence

The results of individual NOS quality appraisals are available in online supplemental figures 1–3. Studies were of good (n=12),^37–39 50 52–56 58 64 65^ fair (n=2)^36 68^ or poor AHRQ standard quality (n=10).^49 51 57 59–63 66 67^ While most cohort studies were of good AHRQ standard quality (n=10),^37–39 50 52–56 58^ all case–control studies were assessed to be of poor quality (n=4).^60–63^ Only one cross-sectional study justified their sample size^65^ and only one case–control study included representative cases.^61^ Due to the nature of cross-sectional^36 64–68^ and case–control studies,^60–63^ they are especially prone to biases (eg, non-response bias, recall bias).^71^

Details of the risk of bias grading across studies using GRADE and outcomes based on this current review are shown in online supplemental table 3. The overall quality of evidence was found to be of very low (PTB, LBW, SGA) and low quality (HDP) due to the observational study designs of the majority of studies and the low individual study quality assessed using NOS (online supplemental figures 1–3).

### Racial discrimination

Racial discrimination was ascertained through several questionnaires and scales which were either previously validated or developed for the specific research study. This included adapted versions of the Experiences of Discrimination Scales (EDS) developed by Krieger *et al* (n=11)^49 54–58 62 63 65–67^ and McNeilly *et al* (n=2),^61 63^ the Racism and Lifetime Experiences Scale (RaLES) (n=2),^38 52^ RaLES Daily Life Experiences Scale (n=2),^37 38^ Racism-Related Experiences Scale (RRE) (n=1),^38^ Everyday Discrimination Scale (EDS) developed by Williams *et al* (n=2),^39 64^ adapted Indigenous Racism Experience questionnaire (n=1),^59^ a single-item question on the frequency of perceived discrimination (n=2)^51 68^ or chronic worry about racial discrimination (n=1),^36^ items on the Growing Up in New Zealand questionnaire (n=1)^53^ and other developed questionnaires assessing racial discrimination (n=2).^50 60^ Some studies focused on the lifetime experience of racism (n=12)^36 38 49 52 54 55 58 61 62 65–67^ while others focused on daily or recent experiences of racism, including during pregnancy or perinatal care (n=12).^37–39 50 51 54 57 59–61 64 68^ Women included in the studies were described to be of different racial and ethnic backgrounds, including black or African-American,^36–39 49 50 52 54–58 60–63 65–68^ Hispanic,^38 39 55 58 68^ non-Hispanic white,^36 49 51 54 57 58 67 68^ Mãori,^53^ Pacific,^53^ Asian,^53 55 58^ Aboriginal,^59^ Romani,^64^ German autochtonous^51^ and Turkish.^51^

### Adverse pregnancy characteristics

Most studies were focused on neonatal adverse pregnancy outcomes, while only one study was focused on a maternal outcome.^58^ Outcomes included PTB (<37 weeks gestation; n=19)^36–39 49–51 53 54 56–59 61 63 65–68^ and gestational age (n=2)^38 67^; LBW (<2500 g; n=7),^49 53 59 61 64 67 68^ very low birth weight (VLBW) (<1500 g; n=3)^60 62 63^ and birth weight for gestational age (BGA) (n=1)^55^; SGA (n=4)^52 58 59 68^; and HDP (n=1).^58^ Only five studies did not report the type of pregnancy,^52 59 64 65 68^ while all other studies included only singleton pregnancies.^36–39 49–51 53–58 60–63 66 67^

Although more than three studies reported LBW,^49 53 59 61 64 67 68^ sufficient information to perform meta-analyses was missing for five studies, even after contact with authors was sought.^49 53 64 67^ The study by Rankin *et al* was not included as it focused solely on preterm LBW births.^61^ From the studies on VLBW,^60 62 63^ two studies contained overlapping participants^60 63^ so no meta-analyses were performed on this outcome. From the 18 studies on PTB,^36–39 49–51 53 54 56–59 61 63 65–67^ five^49 53 58 61 67^ were likewise not included due to insufficient information. The remaining 13 studies reported ORs,^36 38 50 51 54 56 57 59 63 65 66^ PRs^36 37^ and HRs.^38 39^

### Preterm birth (PTB) and gestational age

The most commonly investigated adverse pregnancy outcomes were PTB and gestational age.^36–39 49–51 53 54 56–59 61 63 65–68^ Thirteen studies^36 37 49–51 56–58 61 63 65 67 68^ reported significant positive associations between racial discrimination and PTB and nine studies^37–39 50 53 54 59 61 66^ reported non-significant positive associations. No studies reported significant negative associations (ie, an inverse relationship between experiencing racial discrimination and PTB). Of the studies reporting a significant positive association, 11 were of good or fair quality^36–39 50 53 54 56 58 65 68^ and six of those reported a non-significant association.^37–39 50 53 54^

**Figure 2:**
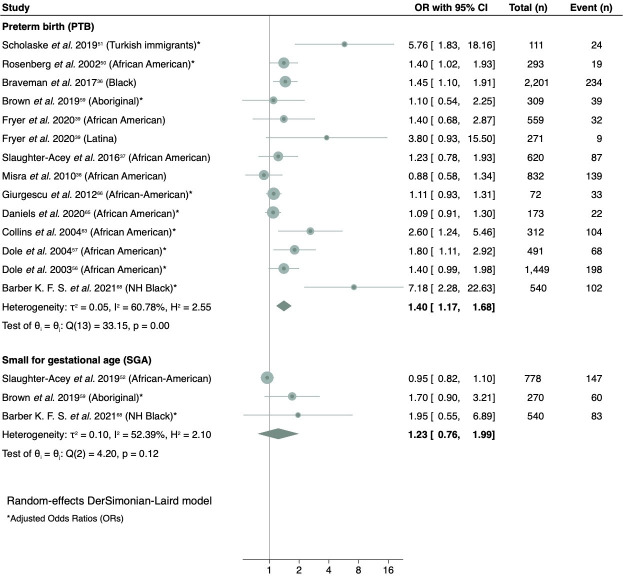
Random-effects DerSimonian–Laird model meta-analyses of the association between racial discrimination and adverse pregnancy outcomes using adjusted ORs.

The meta-analysis included 13 studies^36–39 50 51 54 56 57 59 63 65 66^ comprising nine adjusted^50 51 54 56 57 59 63 65 66^ and four unadjusted associations^36–39^ between racial discrimination and PTB. Overall, data from 9299 participants and 1133 PTB cases were used. A forest plot is shown in figure 2. The overall pooled OR for PTB was 1.40 (95% CI 1.17 to 1.68). Moderate heterogeneity levels were observed (I^2^ = 60.78%) owing to the presence of several influential studies.^65 66 68^ When these studies were excluded, the pooled ORs were attenuated to 1.48 (95% CI 1.20 to 1.82)^65 66^ and OR 1.33 (95% CI 1.13 to 1.55).^68^

A series of sensitivity analyses were conducted. Online supplemental figure 4 shows the results of multiple analyses that exclude one study in each analysis, and the impact of their exclusion on the pooled OR. While excluding individual studies attenuated the overall pooled estimate, no exclusion materially altered the observed pooled association. Pooling crude estimates resulted in an OR of 1.42 (95% CI 1.18 to 1.71) (see online supplemental figure 5). In the subgroup analysis by reported race or ethnicity, an OR of 1.33 (95% CI 1.13 to 1.57) was found when only including results on African-American or black women (see online supplemental figure 6). When using the originally reported measures of association instead of the converted ORs, the pooled OR was attenuated to 1.46 (95% CI 1.20 to 1.77) (see online supplemental figure 7). Exclusion of studies that were graded as low quality in our quality appraisal resulted in a lower but still significant pooled OR of 1.31 (95% CI 1.08 to 1.59) (see online supplemental figure 8).

Asymmetric funnel plots and contour-enhanced funnel plots are shown in online supplemental figure 9, suggesting potential publication bias, poor methodological design of the included studies or true heterogeneity. This asymmetry was only slightly attenuated when excluding low quality studies. The Egger’s test indicated no evidence of small-study effects on summary estimates of PTB (p<0.01). When assuming that there should be a symmetric funnel plot, the trim-and-fill analysis suggests that the number of missing studies was five, and that the adjusted effect estimate is OR 1.20 (95% CI 0.98 to 1.47).

### Low birth weight (LBW), very low birth weight (VLBW) and birth weight for gestational age (BGA)

Other commonly researched outcomes were LBW,^49 53 59 61 64 67^ VLBW^60 62 63^ and BGA.^55^ We were unable to perform meta-analyses on LBW. However, when combined, the studies suggest that women who experienced racial discrimination had a higher risk of a LBW or VLBW infant.^49 53 55 59–64 67^ No studies reported a negative association.^49 53 55 59–64 67^

Both significant positive associations of racial discrimination and LBW^53 59 61 64 67^ and non-significant associations (trending towards positive)^49 53 59 61 67^ were reported. The presence of a significantly higher risk was influenced by the adjusted variables, the severity of racial discrimination experienced and the type of discrimination experienced (lifetime, childhood or recent). Of the studies reporting a significant positive association, two were of good or fair quality^53 64^ and one study reported a non-significant association.^53^

High exposure to racial discrimination compared with low/medium exposure resulted in an OR of preterm LBW infants of 2.5 (95% CI 1.2 to 5.2) for past year experience and 1.5 (95% CI 0.9 to 2.8) for lifetime exposure.^61^ When exploring BGA on a continuous scale, US racial and ethnic minority mothers who reported discrimination in ≥3 domains had lower BGA Z-scores compared with mothers not reporting discrimination in adjusted models (β −0.25, 95% CI −0.45 to –0.04).^55^ In a study among Māori, Pacific and Asian women in New Zealand, the experience of several forms of racial discrimination was a strong predicter of lower birth weight for Māori women only (physical attack: β −1.06 (95% CI −1.83 to –0.28), unfair treatment at work: β −0.95 (95% CI −1.56 to –0.34), in the criminal justice system: β −0.55 (95% CI −1.08 to –0.02) and in the banking system: β −0.73 (95% CI −1.43 to –0.02)).^53^

Similarly, studies exploring VLBW infants of African-American mothers found that perceived racial discrimination was associated with VLBW infants,^60 62 63^ with two studies indicating significant relationships when exposed to ≥1 domains and ≥3 domains of racial discrimination in both unadjusted and adjusted models (participants were asked about racial discrimination across five domains in total: at work, getting a job, at school, getting medical care, getting service at a store or restaurant).^60 63^ While insignificant, ORs of the third study likewise suggest a potential positive association, reporting an unadjusted OR of 1.9 (95% CI 0.5 to 6.6) and adjusted OR of 3.2 (95% CI 0.9 to 11.3).^62^ This association persisted across maternal biomedical, sociodemographic and behavioural characteristics.^63^ None of the studies on birth weight reported an increase in birth weight among those experiencing racial discrimination compared with those who did not.^49 53 59 61 64 67^ Notably, all three studies on VLBW were considered to be of poor quality.

### Small for gestational age (SGA)

Few studies explored SGA in relation to the experience of racial discrimination: two of good quality and one of poor quality.^52 58 59^ Overall, data from 1588 unique participants including 290 SGA cases were used. The overall pooled OR for SGA was 1.23 (95%CI 0.76 to 1.99). Moderate heterogeneity levels were observed (I^2^ =52.39%) (figure 2). When using crude ORs instead of adjusted ORs, the pooled OR was attenuated to 1.68 (95% CI 0.79 to 3.54). Several sensitivity analyses and assessment of publication bias could not be performed due to the limited number of studies.^52 58 59^

Aboriginal women experiencing discrimination in perinatal care were more likely to have an SGA infant than Aboriginal women not experiencing racial discrimination when adjusting for parity, stressful events and social health issues during pregnancy (OR 1.7, 95% CI 0.9 to 3.21) or when adjusting for parity and cigarette/cannabis use (OR 1.9, 95% CI 1.0 to 3.5).^59^ When assessing overall racism experienced by African-American women, this was not associated with SGA (OR 0.95, 95% CI 0.82 to 1.10).^52^ However, stratified by age, African-American women aged >25 years experiencing racism were more likely to deliver a SGA infant (OR 1.45, 95% CI 1.02 to 2.08) than women not experiencing racism.^52^ In contrast, racism was not associated with SGA in adolescents aged ≤18 years (OR 0.92, 95% CI 0.66 to 1.28) or emerging adults aged 19–24 years (OR 0.86, 95% CI 0.69 to 1.06),^52^ and was not the explaining factor in the disparity between non-Hispanic black women and non-Hispanic white women.^58^

### Hypertensive disease of pregnancy (HDP)

Only one (good quality) study reported on HDP. Among the large and geographically diverse cohort of nulliparous women, non-Hispanic black women were more likely to experience HDP than non-Hispanic white women. However, this disparity was not explained by differences in self-reported racism (adjusted OR 0.95, 95% CI 0.79 to 1.14).^58^

## Discussion

This systematic review and meta-analysis assessed self-perceived racial discrimination and adverse pregnancy outcomes. While acknowledging the overall low quality evidence, these study results suggest that there may exist a positive relationship between increased racial discrimination and worse pregnancy outcomes. Across 24 included publications, both significant positive associations^36 37 49–53 56–61 63–65 67 68^ and non-significant positive associations were reported.^37–39 49 50 52–54 58 59 61 62 66 67^ No studies reported significant negative associations. The overall pooled OR was 1.40 (95% CI 1.17 to 1.68; 13 studies) for PTB, with an OR of 1.31 (95% CI 1.08 to 1.59) when excluding low quality studies, and 1.23 (95% CI 0.76 to 1.99; 3 studies) for SGA. Across sensitivity and subgroup analyses, the ORs slightly attenuated but remained indicative of a significant positive association.

When assessing both subjective (self-reported) and objective outcomes in cross-sectional and longitudinal studies, consistent associations with poor health across a range of outcomes have been reported.^69 72^ Our findings align with existing evidence on perceived racial discrimination as an important risk factor for adverse pregnancy outcomes. A 2011 integrative review (10 studies) by Giurgescu *et al* found consistent positive relationships with LBW, PTB and VLBW.^73^ Moreover, Alhusen *et al* found racial discrimination to be a significant risk factor for LBW, PTB and SGA in their integrative review of 15 studies.^25^ Finally, a small narrative review by Mutambudzi *et al* argued that factors associated with racial discrimination accounted for some of the racial disparities in LBW and PTB.^74^

Pervasive in people’s day-to-day lives, racism has far-reaching implications on the experiences of racialised individuals. As an upstream factor, it shapes other social determinants of health such as employment, poverty, education and housing. Relating more directly to health, racism can impact what services and resources are available, such as referral to specialist care, access to health insurance and access to public health services.^75^ Several individual- and context-level factors may mediate or moderate the relationship between racial discrimination and pregnancy outcomes. For example, some studies indicated that the significant impact of racism on PTB was moderated by depressive symptomatology.^38^ Likewise, others found that smoking mediated 13.5% of the total effect of self-reported everyday discrimination and LBW.^64^ However, the majority of reviewed studies did not explore moderating or mediating factors. Broader literature suggests that factors such as racial or ethnic identification, positive racial evaluation, positive in-group racial attitudes, meritocratic world views, generic coping strategies and social support may reduce the health impacts of discrimination.^76^ Mechanistic pathways through which racial discrimination adversely impacts pregnancy outcomes have yet to be elucidated. An American study of 107 921 women found that significantly higher proportions of racialised women had discontinuous insurance coverage between preconception and delivery compared with white women. The resulting racial-ethnic disparities in access to preconception, prenatal and postpartum care may lead to increased adverse pregnancy outcomes in racialised populations.^77^ To be able to address racial discrimination and its health impacts more comprehensively, these factors and mechanistic pathways require further exploration.

Moreover, a long history of systemic discrimination, bias, direct racism and associated trauma resulting in psychosocial stress, high-effort coping strategies and distrust of the medical system culminate in poorer health outcomes and shorter life expectancies among racialised communities.^78–80^ Illustratively, increased allostatic load (a marker of chronic physiologic stress experienced over a life course), inflammation and oxidative stress have been linked to inequities like racism and discrimination. Evidence suggests that chronic psychological stress may accelerate telomere shortening and cellular ageing, which are associated with the onset of disease, thereby connecting the experience of prolonged racism to tangible disease outcomes, including communicable and non-communicable diseases.^81 82^ Emerging research on anticipating discrimination suggests that the chronic activation of cognitive imagery of a stressor itself can further result in prolonged stress and negative health impacts.^83^ Recent studies report that chronic worry about racial discrimination may explain the persistent disparities in pregnancy outcomes through neuroendocrine, vascular, inflammatory and immune processes involved in both stress responses and parturition.^36^

Early exposure to stress (preconception or in utero) and adverse effects on fetal and neonatal health may predispose individuals to chronic diseases in later life, while epigenetic mechanisms may have intergenerational health consequences. Illustratively, women with a history of PTB or SGA tend to have a higher allostatic load than women giving birth to term and normal weight infants.^84^ Discriminatory events often begin early in childhood and continue through adolescence and adulthood, resulting in an accumulation of discriminatory stressors over the life course, both before and during pregnancy, which may impact pregnancy outcomes. However, thus far, limited attention has focused on capturing cumulative exposure to discrimination over the life course, with most studies focusing instead on recent occurrences.^69^ Furthermore, it is important to note that, while the stress of daily life may result from racial discrimination, this effect can be magnified by coexisting correlates of socioeconomic status, which may explain differences in health outcomes among black women of differing socioeconomic status.^85^ This may also shed light on the worsening health disparities between white women in the USA compared with women in other countries which have lower levels of income inequality and higher expenditures on social supports.^23^

### Strengths and limitations

This study has several limitations. First, perceived or self-reported racial discrimination is only a subset of total racial discrimination experienced by marginalised communities.^86^ Subtle forms of racial discrimination may be perpetuated in normative forms beyond people’s conscious awareness, resulting from legitimising ideologies and perceived power that justify the status quo.^87^ This includes established forms of systematic, institutional and organisational racism, or internalised racism, which may not always be perceived or self-reported as racial discrimination but nonetheless impacts health outcomes.^88^ Importantly, other privileged or marginalised group identities, such as high or low socioeconomic status, respectively, may differentially shape the impacts of racial discrimination on health while also being differentially shaped by racial discrimination. For instance, while low socioeconomic status among racialised groups likely contributes to worse outcomes, the underlying structural/systemic racism is what leads to the racialised poverty. However, current research frequently fails to recognise these intersectionalities.^69^ Furthermore, in the included studies, experiences of discrimination were only considered for an individual’s self-identified, but not socially perceived, race or ethnicity, which has been shown to correlate to differential health outcomes among members of the same race or ethnicity. In addition, measurements of perceived discrimination may be influenced by perception biases such as minimising bias (leading to under-reporting) or vigilance bias (leading to over-reporting).^86 89^ Nonetheless, although perceived racism does not stand alone as an exposure, it is useful as a standardised and validated marker for the true, complex, multifaceted experience of racism across the life course in order to measure one level of the exposure to racism. Moreover, regardless of the ‘objectivity’ of such racial discrimination reporting, the subjective experience of racial discrimination may impact health across racial and ethnic groups.^89 90^

Second, most included studies were based in the USA and few included marginalised racial or ethnic groups beyond African-Americans.^36–39 49 50 52 54–58 60–63 65–68^ Likewise, most of the racial discrimination scales used were developed in the USA and focused mainly on the African-American experience. These instruments may not be fully able to capture all forms of discriminatory experiences across different marginalised racial and ethnic groups,^91^ thereby limiting the generalisability of our results across different ethnogeographic and cultural settings. Third, the available evidence was limited and of relatively low quality, precluding a more robust synthesis and further detailed meta-analyses (including subgroup and sensitivity analysis). Heterogeneity was noted in both meta-analyses, which may have arisen from different levels of adjustment, different tools used to assess racial discrimination, and varied sample sizes, study qualities and time periods. Likewise, heterogeneity was found within studies when different models, discriminatory behaviours or subgroups were described, leading to both significant positive associations and non-significant associations within the same study. Importantly, the funnel plot asymmetry may be a potential indication of negative studies missing (publication bias) or could be an indication of true heterogeneity.^92^ Under the assumption that there should be a symmetric funnel plot, the trim-and-fill analysis suggested that the pooled effect estimate reduced to OR 1.20 (95% CI 0.98 to 1.47). High quality studies that allow for detailed analysis across population groups are therefore needed to further confirm the results found in this systematic review. Fourth, while some scales used were validated within studies, the validity of other scales was rarely examined outside of the scale developers.^91^ Some of the retrieved studies based the experience of racial discrimination on only one question in a broader survey without validation, raising concerns about the reliability of the data generated.^36 51 53 68^ Lastly, to limit potential bias and confounders and ensure replicability, we only searched peer-reviewed academic journals. As a result, available evidence reported in the grey literature may have been missed, and the studies retrieved may have been impacted by publication bias.

Despite this, our review has several strengths, including a broad definition of adverse pregnancy outcomes allowing for a wide-ranging examination of the impacts of racial discrimination, a detailed comprehensive search strategy to gather available evidence and no restrictions on date or language of publication.

### Interventions

The World Health Organization highlights the global need to address the impact of racial discrimination, racism and related intolerance on health.^93^ There is a remarkable body of evidence identifying the negative impacts of racial discrimination on health, and several interventions have been suggested to reduce these negative health impacts. These include, for example, antiracism counter marketing, values affirmation and forgiveness interventions.^69^ Some of the approaches that can be taken to reduce racial inequalities in health outlined by Williams *et al* include: (1) creating communities of opportunity that could minimise structural racism; (2) ensuring the emphasis on ‘health for all’ and public health approaches in healthcare systems, increasing the diversity of health professionals and ensuring that patients’ social needs are addressed as part of their management; and (3) building political will and support to counteract social and health inequities.^94^ Studies have proposed group prenatal care as an alternative prenatal care delivery model to improve pregnancy outcomes, particularly among black women.^95 96^ This model is thought to increase social support and lead to stronger physician–patient relationships. Moreover, it is critical to work towards decolonising and improving medical training by universally removing well-documented examples of racial bias which continue to perpetuate health inequities. This includes the lack of teaching on dermatology and differential disease presentations in non-white individuals, inaccuracies in pulse oximetry technology, unsubstantiated race-based adjustments to measuring renal function, and inadequate teaching around individual biases and the social drivers of health inequities.^97–99^

Additionally, there is a need for more systematic population-based assessments of racial discrimination to collect data on maternal exposures and outcomes in the perinatal period. Such large-scale surveillance systems, implemented across diverse populations, can play a crucial role in closing the racial/ethnic gap in fetal, neonatal and maternal health outcomes.

Importantly, efforts to counter racial discrimination must focus on systemic policy changes rather than individual-level intervention and prevention efforts to create sustainable change. In 2019, NHS England committed to ensuring that, by 2024, 75% of women from black, Asian and minority ethnic communities will receive continuity of care from their midwife throughout the perinatal period.^100^ Researchers have also called for reparations to historically oppressed communities to counter the discriminatory distribution of resources and increase access to health-producing resources.^101^ Similarly, a 2018 study found that an unconditional prenatal income supplement for low-income women was associated with reduced disparities in adverse birth outcomes including LBW, PTB and breastfeeding initiation.^102^ Dismantling the structures and policies that enable institutional and interpersonal racism, underlying racial and/or ethnic disparities in health and intersecting social inequalities is essential to improve overall health in societies. Partnerships with community-based reproductive justice and women’s health organisations who work in this area are indispensable in improving health for racialised women in a community-centred way.

Finally, it is important to acknowledge and recognise that modern-day settler colonialism, apartheid, xenophobia, islamophobia and antisemitism exist as forms of contemporary racism that are on the rise, both directly and indirectly contributing to negative health outcomes on a large scale. Historical and current efforts to highlight and boycott racist countries, policies and politicians for their roles in engineering racism serve as concrete interventions aiming to deconstruct racist structures on a global level. Examples of such efforts include the civil rights movement in the USA, the boycott of apartheid South Africa, and the efforts to mark and fight apartheid in Palestine, as well as efforts to frame anti-islamophobia in India and elsewhere. Globally, it is critical for public and global health scholars, educators and practitioners to research and fight these phenomena to contribute to better and sustainable health outcomes at the population level.

## Conclusions

Our study highlights that racial discrimination has adverse impacts on pregnancy outcomes, with the greatest evidence found for PTB. While this work highlights the importance of research on racial discrimination and health, broader questions about limitations of this field of quantitative research remain. By reducing complex social interactions and experiences to quantitative findings alone, which may be limited by the tools used to take measurements, we risk missing nuances by focusing on statistical significance over the reported experiences of affected individuals. Qualitative studies further exploring these complex social interactions may be able to specifically identify occurrences of racial discrimination, macrolevel biases and implicit biases, address the larger sentiments of society around race and racism, and further push for changes in outdated medical and public health curricula to reflect the advances in our understanding of the relationships between race, racism and health. To further explore racial discrimination and their underlying mechanisms (including mediating and moderating factors), there is a need for higher quality quantitative and qualitative evidence using a life course approach from large ethnographically diverse cohorts that assess different forms, levels and contexts of racial discrimination on a plethora of fetal, neonatal and maternal health outcomes.

## Data Availability

All data relevant to the study are included in the article or uploaded as supplementary information.
