# Supplementary material for: Racial discrimination and adverse pregnancy outcomes: a systematic review and meta-analysis
Source: BMJ Glob Health. 2022 Jun 27;7(8):e009227. doi: 10.1136/bmjgh-2022-009227 (PMC9344988; doi:10.1136/bmjgh-2022-009227)
Supplement: Supplementary data [file bmjgh-2022-009227supp001.pdf]

BMJ Publishing Group Limited (BMJ) disclaims all liability and responsibility arising from any reliance placed on this supplemental material which has been supplied by the author(s)

Racial discrimination and adverse pregnancy outcomes: a systematic review and meta-analysis

Supplementary Material

van Daalen KR, *et al. BMJ Global Health* 2022; 7:e009227. doi: 10.1136/bmjgh-2022-009227

Supplemental figure 1. Quality assessment scores of included cross-sectional studies (n = 6) using the Newcastle Ottawa Scale[37]

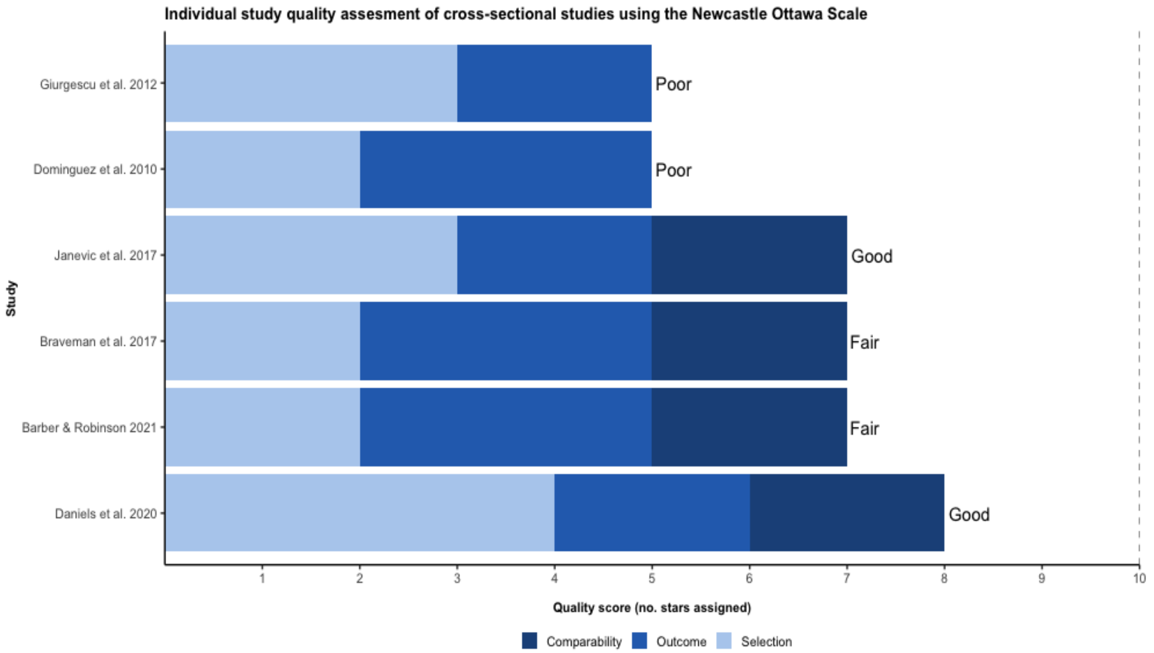

Each point represents a point for a given quality indicator. A maximum of 10 points can be awarded.
AHRQ, Agency for Healthcare Research and Quality; quality categorized as good, fair, or poor.

Thresholds for converting the Newcastle-Ottawa scales to AHRQ standards (good, fair, and poor):
Good quality: 3 or 4 stars in selection domain AND 1 or 2 stars in comparability domain AND 2 or 3 stars in outcome/exposure domain
Fair quality: 2 stars in selection domain AND 1 or 2 stars in comparability domain AND 2 or 3 stars in outcome/exposure domain
Poor quality: 0 or 1 star in selection domain OR 0 stars in comparability domain OR 0 or 1 stars in outcome/exposure domain

Supplemental figure 2. Quality assessment scores of included case-control studies (n = 4) using the Newcastle Ottawa Scale[37]

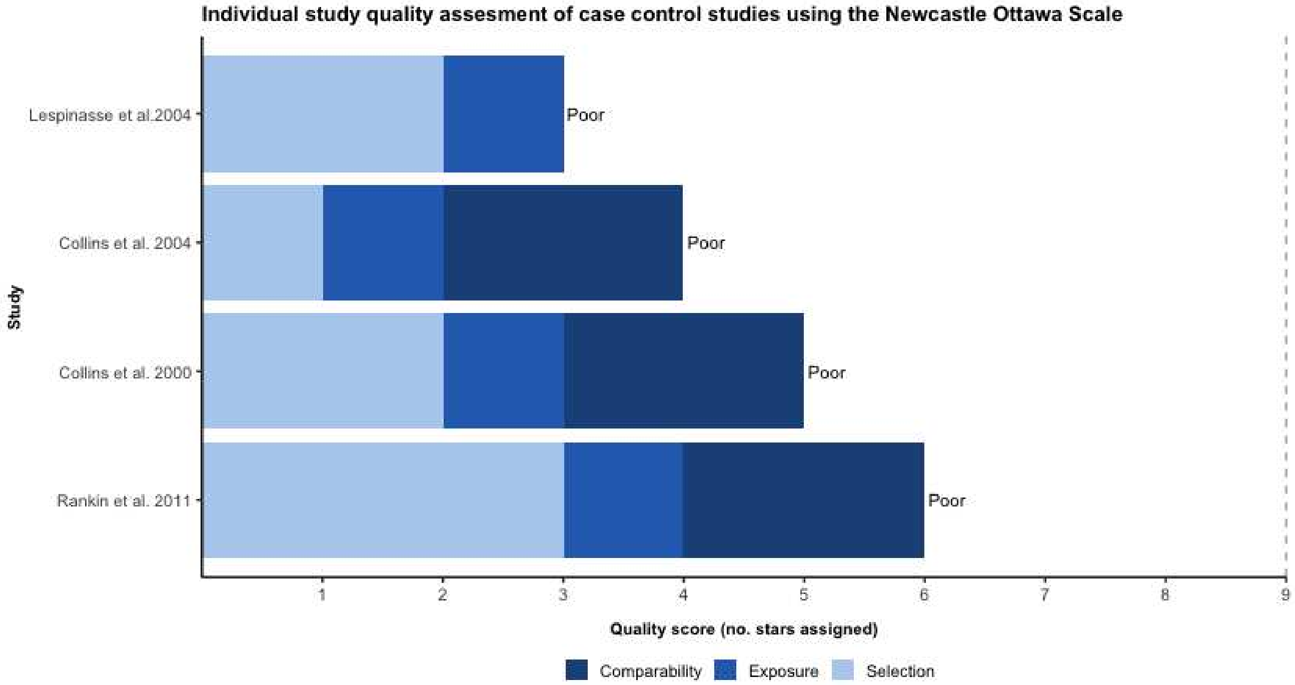

Each point represents a point for a given quality indicator. A maximum of 9 points can be awarded.
AHRQ, Agency for Healthcare Research and Quality; quality categorized as good, fair, or poor.

Thresholds for converting the Newcastle-Ottawa scales to AHRQ standards (good, fair, and poor):
Good quality: 3 or 4 stars in selection domain AND 1 or 2 stars in comparability domain AND 2 or 3 stars in outcome/exposure domain
Fair quality: 2 stars in selection domain AND 1 or 2 stars in comparability domain AND 2 or 3 stars in outcome/exposure domain
Poor quality: 0 or 1 star in selection domain OR 0 stars in comparability domain OR 0 or 1 stars in outcome/exposure domain

Supplemental figure 3. Quality assessment scores of included cohort studies (n = 14) using the Newcastle Ottawa Scale[37]

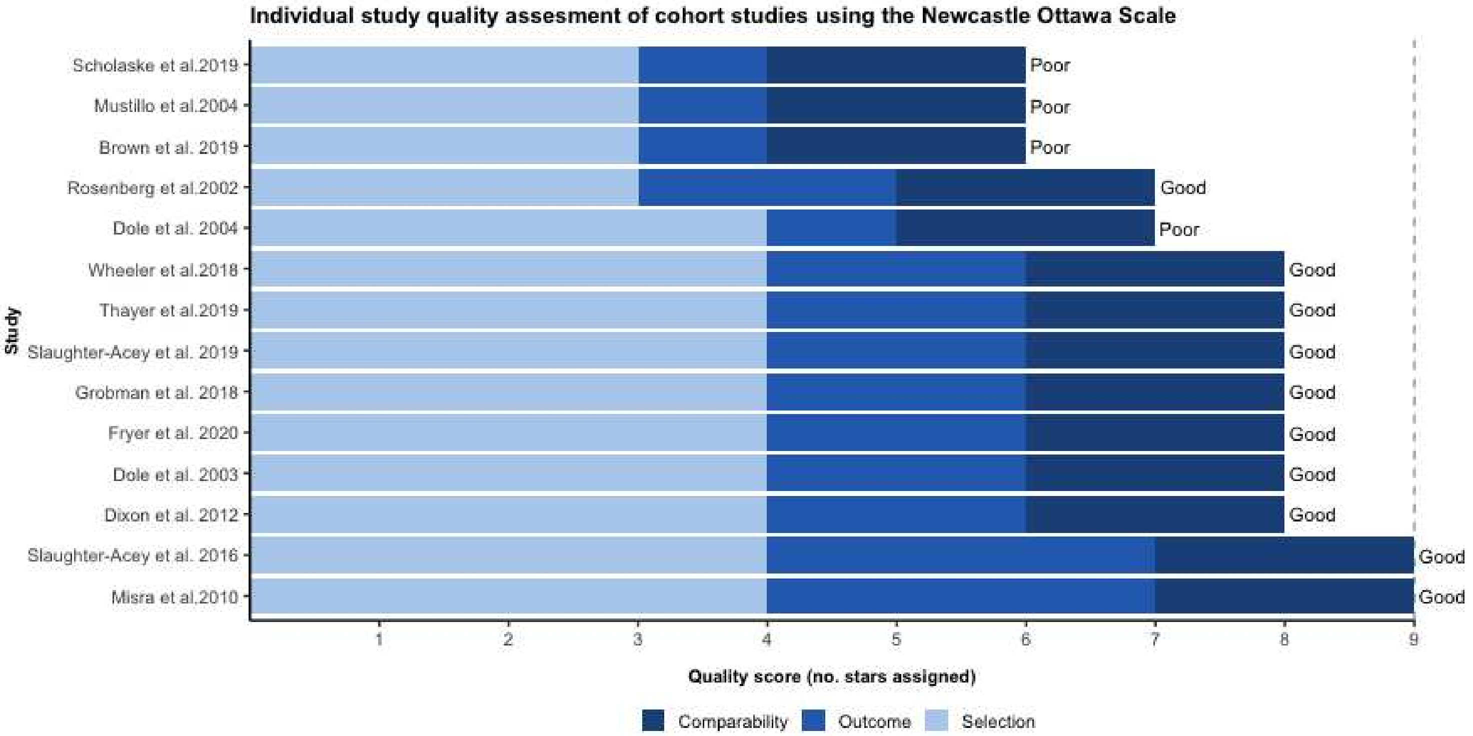

Each point represents a point for a given quality indicator. A maximum of 9 points can be awarded.
AHRQ, Agency for Healthcare Research and Quality; quality categorized as good, fair, or poor.

Thresholds for converting the Newcastle-Ottawa scales to AHRQ standards (good, fair, and poor):
Good quality: 3 or 4 stars in selection domain AND 1 or 2 stars in comparability domain AND 2 or 3 stars in outcome/exposure domain
Fair quality: 2 stars in selection domain AND 1 or 2 stars in comparability domain AND 2 or 3 stars in outcome/exposure domain
Poor quality: 0 or 1 star in selection domain OR 0 stars in comparability domain OR 0 or 1 stars in outcome/exposure domain

Omitted study	OR with 95% CI	P-value
Scholaske *et al.* 2019[51] (Turkish immigrants)*	1.34 [1.14, 1.58]	0.000
Rosenberg *et al.* 2002[50] (African American)*	1.41 [1.16, 1.72]	0.001
Braveman *et al.* 2017[36] (Black)	1.41 [1.16, 1.71]	0.001
Brown *et al.* 2019[59] (Aboriginal)*	1.42 [1.18, 1.71]	0.000
Fryer *et al.* 2020[39] (African American)	1.41 [1.17, 1.69]	0.000
Fryer *et al.* 2020[39] (Latina)	1.38 [1.15, 1.64]	0.000
Slaughter-Acey *et al.* 2016[37] (African American)	1.43 [1.18, 1.73]	0.000
Misra *et al.* 2010[38] (African American)	1.46 [1.21, 1.76]	0.000
Giurgescu *et al.* 2012[66] (African-American)*	1.48 [1.20, 1.82]	0.000
Daniels *et al.* 2020[65] (African American)*	1.48 [1.20, 1.82]	0.000
Collins *et al.* 2004[63] (African American)*	1.35 [1.14, 1.61]	0.001
Dole *et al.* 2004[57] (African American)	1.37 [1.14, 1.65]	0.001
Dole *et al.* 2003[56] (African American)*	1.41 [1.16, 1.72]	0.001
Barber K. F. S. *et al.* 2021[68] (NH Black)*	**1.33 [1.13, 1.55]**	0.000

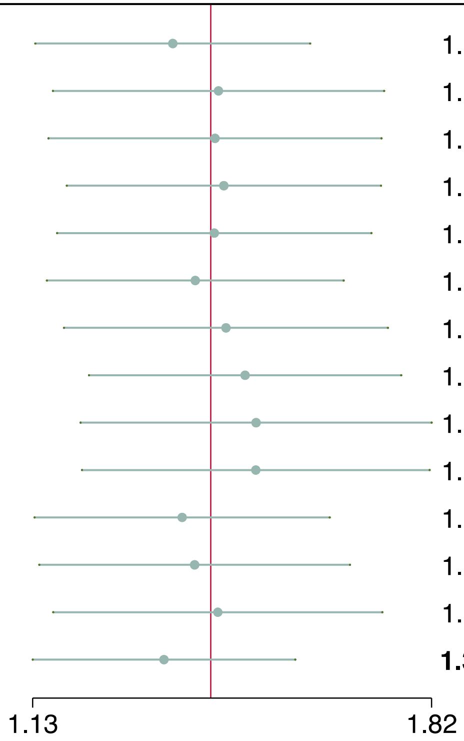

DerSimonian-Laird model
*Adjusted Odds Ratios (ORs)

Supplement figure 4. Leave-one-out random-effects DerSimonian-Laird model meta-analyses of the association between racial discrimination and preterm birth outcomes that looked at the impact of excluding each study from the meta-analysis in turn.

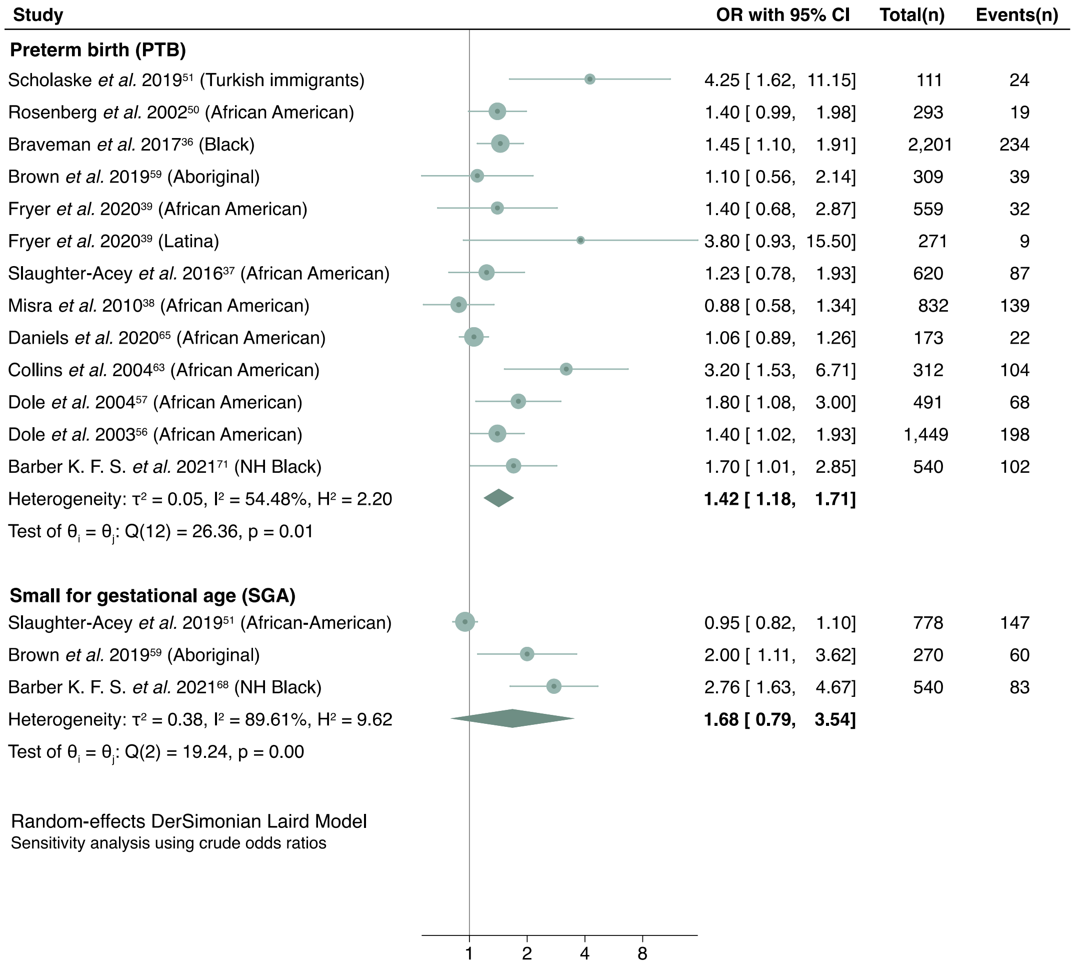

Supplement figure 5. Random-effects DerSimonian-Laird model meta-analyses of the association between racial discrimination and adverse pregnancy outcomes using crude odds ratios.

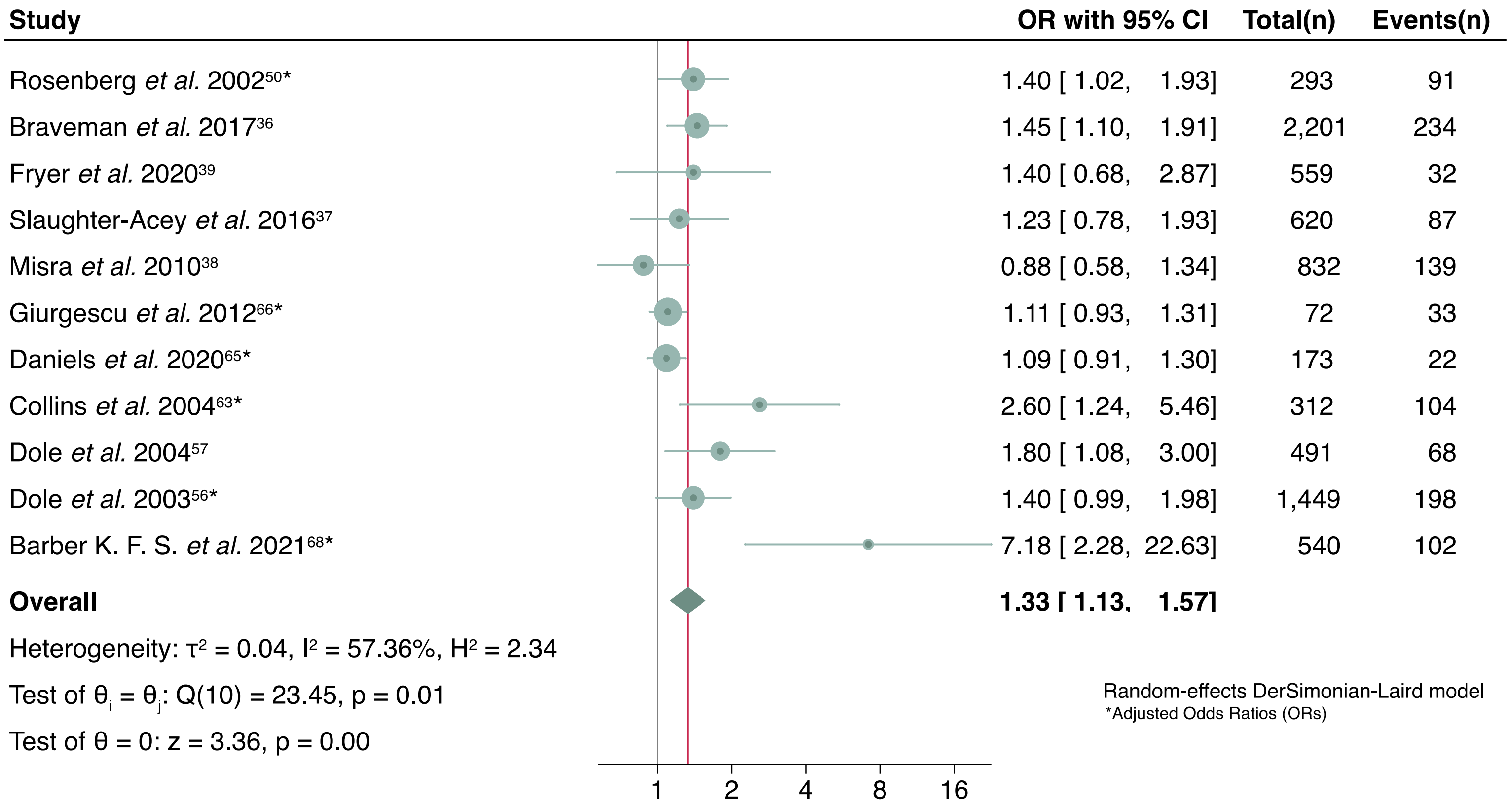

Supplement figure 6. Random-effects DerSimonian-Laird model meta-analysis of the association between racial discrimination and preterm birth among African American women.

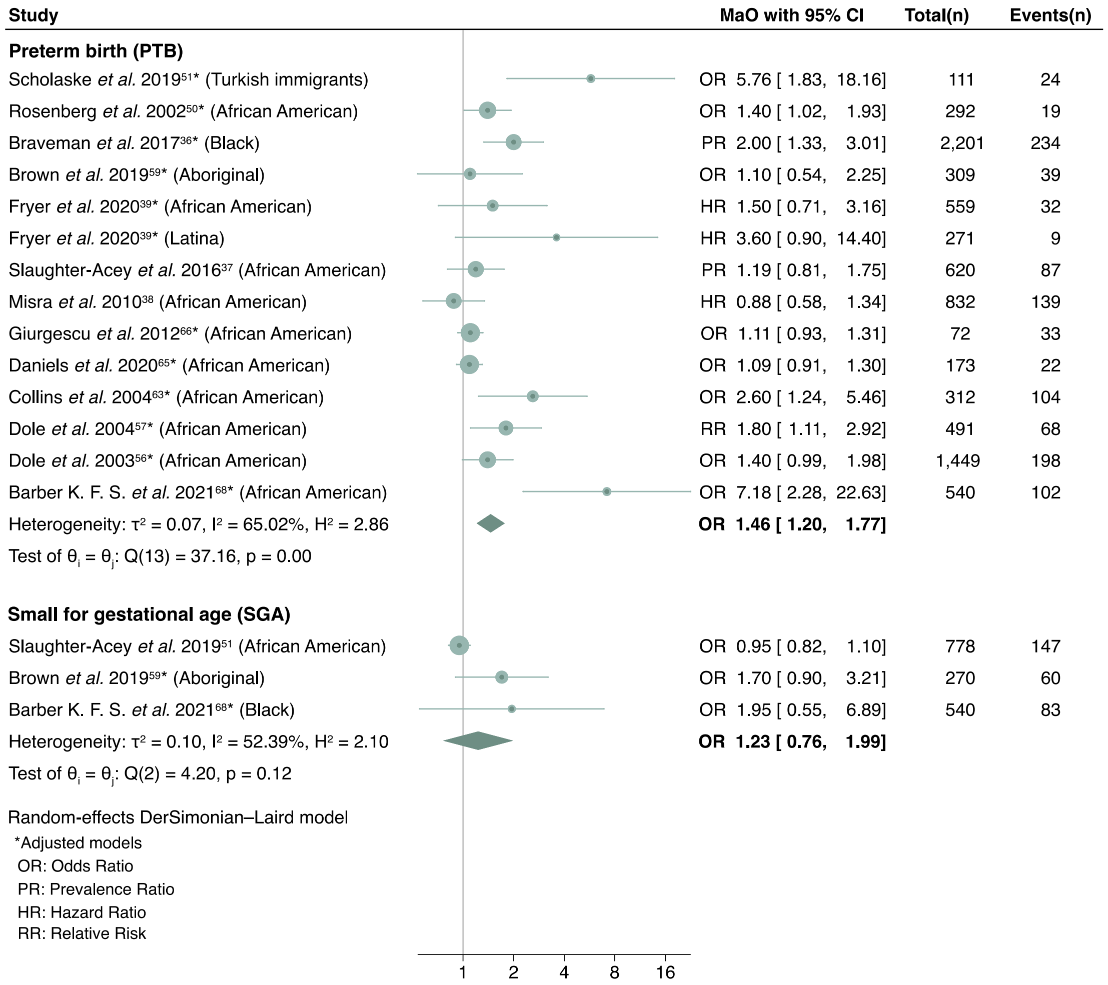

Supplement figure 7. Random-effects DerSimonian-Laird model meta-analysis of the association between racial discrimination and adverse pregnancy outcomes using originally reported measures of association (MoA).

 BMJ Publishing Group Limited (BMJ) disclaims all liability and responsibility arising from any reliance placed on this supplemental material which has been supplied by the author(s)

Study	OR with 95% CI	Total(n)	Events(n)
Rosenberg *et al.* 2002[50] (African American)*	1.40 [1.02, 1.93]	292	19
Braveman *et al.* 2017[36] (Black)	1.45 [1.10, 1.91]	2,201	234
Brown *et al.* 2019[59] (Aboriginal)*	1.10 [0.54, 2.25]	309	39
Fryer *et al.* 2020[39] (African American)	1.40 [0.68, 2.87]	559	32
Fryer *et al.* 2020[39] (Latina)	3.80 [0.93, 15.50]	271	9
Slaughter-Acey *et al.* 2016[37] (African American)	1.23 [0.78, 1.93]	620	87
Misra *et al.* 2010[38] (African American)	0.88 [0.58, 1.34]	832	139
Daniels *et al.* 2020[65] (African American)*	1.09 [0.91, 1.30]	72	33
Dole *et al.* 2003[56] (African American)*	1.40 [0.99, 1.98]	491	68
Barber K. F. S. *et al.* 2021[68] (NH Black)*	7.18 [2.28, 22.63]	540	102
**Overall**	**1.31 [1.08, 1.59]**		

Heterogeneity: $\tau^2 = 0.04$, $I^2 = 51.21\%$, $H^2 = 2.05$

Test of $\theta_i = \theta_j$: Q(9) = 18.45, p = 0.03

Test of $\theta = 0$: z = 2.79, p = 0.01

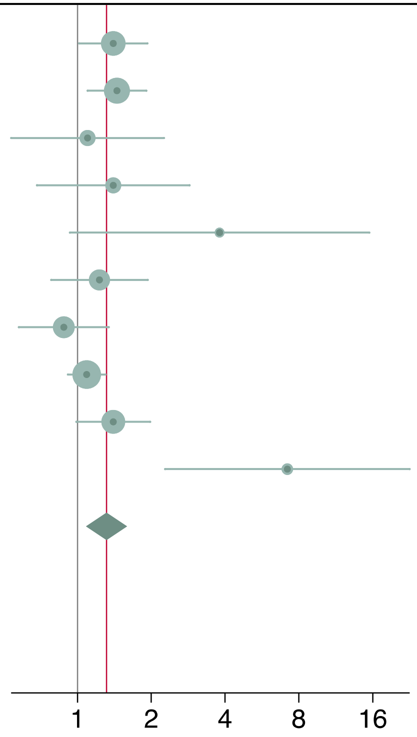

Random-effects DerSimonian–Laird model
* Adjusted models

Supplement figure 8. Random-effects DerSimonian-Laird model meta-analysis of the association between racial discrimination and adverse pregnancy outcomes using studies assessed to be of good or fair quality.

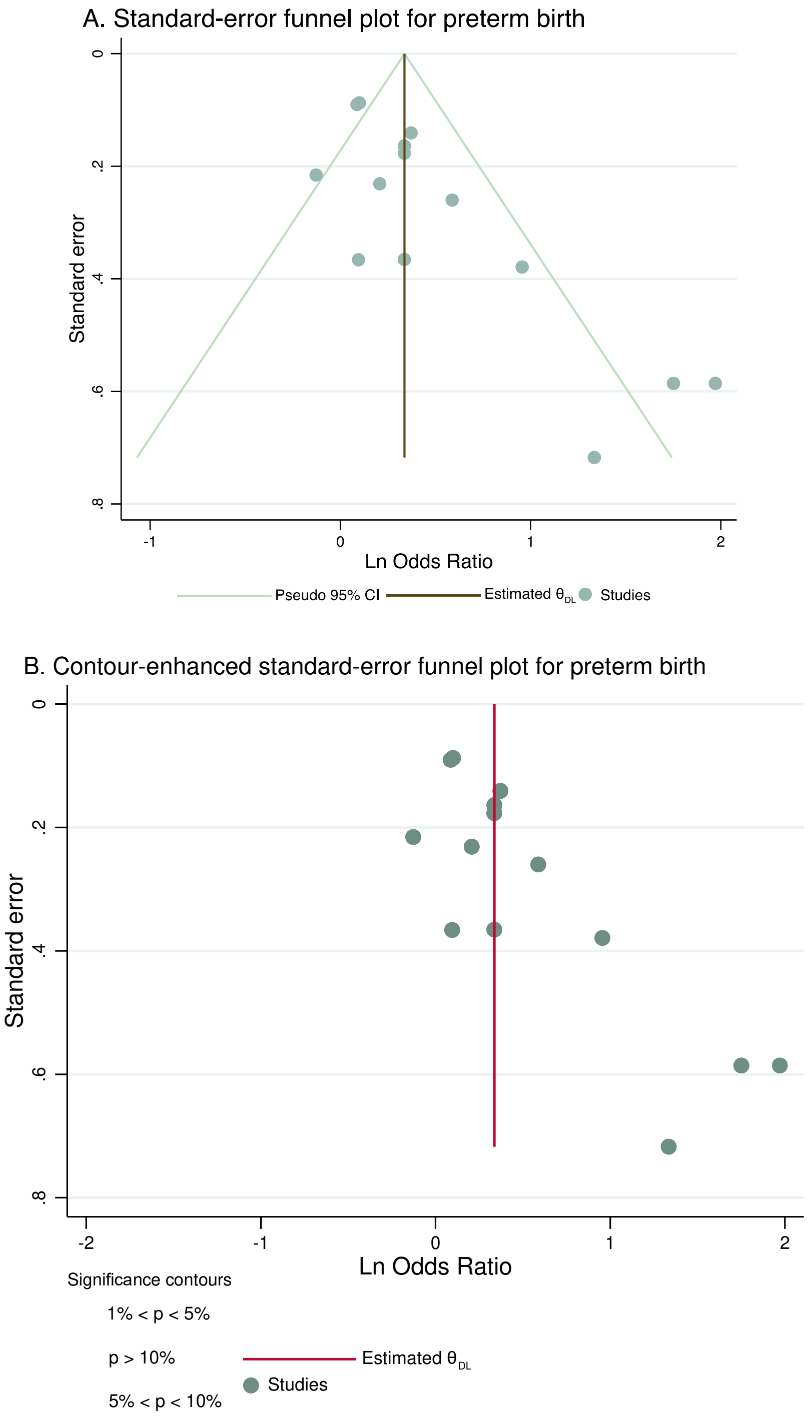

Supplement figure 9. Funnel plots to assess publication bias on studies exploring the association between racial discrimination and adverse pregnancy outcomes.

Supplemental table 1. Full Search Strategy

Database	
Ovid MEDLINE	1 exp pregnant women/ or exp Pregnancy/ or Pregnan*.ti,ab. or Childbearing.ti,ab. or "Child bearing".ti,ab. or Pregnan*.kw. or Childbearing.kw. or "Child bearing".kw. or gestation*.ti,ab. or gestation*.kw. or Placenta*.kw. or Placenta*.ti,ab. or parturition.kw. or parturition.ti,ab. or expectant.ti,ab. or expectant.kw. (1112433)<br>2 exp racism/ or racism.ti,ab. or racism.kw. or racist.ti,ab. or racist.kw. (6086)<br>3 (raci* or race* or ethnic* or cultur*).ti,ab. or exp minority groups/ or exp ethnic groups/ or exp Continental Population Groups/ or raci*.kw. or race*.kw. or ethnic*.kw. or cultur*.kw. (1610446)<br>4 exp prejudice/ or exp social discrimination/ or exp bullying/ or discrim*.ti,ab. or hostil*.ti,ab. or prejud*.ti,ab. or harass*.ti,ab. or oppress*.ti,ab. or bully*.ti,ab. or bias.ti,ab. or discrim*.kw. or hostil*.kw. or prejud*.kw. or harass*.kw. or oppress*.kw. or bully*.kw. or bias.kw. or injustic*.ti,ab. or injustic*.kw. (487641)<br>5 1 and (2 or (3 and 4)) (1800)
Embase	1 exp *pregnant women/ or exp *Pregnancy/ or Pregnan*.ti,ab. or Childbearing.ti,ab. or "Child bearing".ti,ab. or Pregnan*.kw. or Childbearing.kw. or "Child bearing".kw. or gestation*.ti,ab. or gestation*.kw. or Placenta*.kw. or Placenta*.ti,ab. or parturition.kw. or parturition.ti,ab. or expectant.ti,ab. or expectant.kw. (883863)<br>2 exp *racism/ or racism.ti,ab. or racism.kw. or racist.ti,ab. or racist.kw. (6180)<br>3 exp *prejudice/ or exp *bullying/ or discrim*.ti,ab. or hostil*.ti,ab. or prejud*.ti,ab. or harass*.ti,ab. or oppress*.ti,ab. or bully*.ti,ab. or bias.ti,ab. or discrim*.kw. or hostil*.kw. or prejud*.kw. or harass*.kw. or oppress*.kw. or bully*.kw. or bias.kw. or injustic*.ti,ab. or injustic*.kw. (583433)<br>4 (raci* or race* or ethnic* or cultur*).ti,ab. or exp *race/ or exp *racial difference/ or exp *ethnicity/ or exp *minority group/ or exp *ethnic group/ or raci*.kw. or race*.kw. or ethnic*.kw. or cultur*.kw. (1826140)<br>5 1 and (2 or (3 and 4)) (1544)

CINAHL

S7	S3 and (s4 or (s5 and s6))
S6	((MH "Race Factors") OR (MH "Ethnic Groups+") OR (MH "Minority Groups")) OR ((raci* or race* or ethnic* or culture* or minorit*))
S5	((MH "Prejudice+") OR (MH "Cultural Bias") OR (MH "Discrimination+") OR (MH "Bullying+")) OR ((Prejudic* or discrim* or hostil* or harass* or oppress* or bully* or bias* or injusti*))
S4	(MH "Racism") or (racism or racist)
S3	S1 OR S2
S2	(pregnan* or childbearing or "child bearing" or gestation* or placenta* or parturition or expectant)
S1	(MH "Pregnancy+") OR (MH "Expectant Mothers")

PsycINFO

S14	S3 AND S12
S13	#s1 and #s2
S12	S4 OR S11
S11	S7 AND S10
S10	S8 OR S9
S9	(raci* or race* or ethnic* or culture* or minorit*)

S8	(DE "Ethnic Identity" OR DE "Multiracial") OR (DE "Racial and Ethnic Groups" OR DE "African Cultural Groups" OR DE "Arabs" OR DE "Asians" OR DE "Blacks" OR DE "European Cultural Groups" OR DE "Indigenous Populations" OR DE "Latinos/Latinas" OR DE "Romanies" OR DE "Tribes" OR DE "Whites")
S7	S5 OR S6
S6	(Prejudic* or discrim* or hostil* or harass* or oppress* or bully* or bias* or injusti*)
S5	((DE "Prejudice" OR DE "Religious Prejudices") OR (DE "Discrimination" OR DE "Cognitive Discrimination" OR DE "Discrimination Laws" OR DE "Drug Discrimination" OR DE "Perceptual Discrimination" OR DE "Social Discrimination" OR DE "Stimulus Discrimination")) OR (DE "Bullying" OR DE "Cyberbullying")
S4	DE "Racism" or DE "Race and Ethnic Discrimination" OR DE "Racial Disparities" or (racism or racist)
S3	S1 OR S2
S2	(pregnan* or childbearing or "child bearing" or gestation* or placenta* or parturition or expectant)
S1	(DE "Pregnancy" OR DE "Adolescent Pregnancy" OR DE "Pregnancy Outcomes" OR DE "Primipara") OR (DE "Expectant Mothers")

SOCINDEX

S7	S3 and (s4 or (s5 and s6))
S6	(DE "RACE" OR DE "BLACK race" OR DE "RACIAL minorities" OR DE "RACIALIZATION" OR DE "MINORITIES" OR DE "ATTITUDES of ethnic groups" OR DE "MINORITY families" OR DE "MINORITY parents" OR DE "MINORITY women" OR DE "RACIAL minorities" OR DE "RELIGIOUS minorities" OR DE "ETHNIC groups" OR DE "AFRICAN Americans" OR DE "ARABS" OR DE "ARCTIC peoples" OR DE "ASIAN Americans" OR DE "ASIANS" OR DE "ETHNIC groups in mass media" OR DE "ETHNIC relations" OR DE "ETHNOLINGUISTIC groups" OR DE "EUROPEANS" OR DE "HISPANIC Americans" OR DE "INDIGENOUS peoples" OR DE "INDIGENOUS peoples of the Americas" OR DE "RACIALLY mixed people" OR DE "MEDICAL care of ethnic groups") or (raci* or race* or ethnic* or culture* or minorit*)
S5	(DE "TRANSCULTURAL medical care" OR DE "DISCRIMINATION in medical care" OR DE "MEDICAL care of minorities" OR DE "HEALTH disparities" OR DE "PREJUDICES" OR DE "CULTURAL prejudices" OR DE "DISCRIMINATION" OR DE "COVERT discrimination" OR DE "PERCEIVED discrimination" OR DE "INDIRECT discrimination" OR DE "PERCEIVED discrimination" OR DE "BULLYING") or (Prejudic* or discrim* or hostil* or harass* or oppress* or bully* or bias* or injusti*)
S4	(DE "RACISM" OR DE "INSTITUTIONAL racism" OR DE "RACE discrimination" OR DE "RACE discrimination in medical care" OR DE "HEALTH & race" OR DE "MORTALITY & race) or (racism or racist)
S3	S1 OR S2
S2	(pregnan* or childbearing or "child bearing" or gestation* or placenta* or parturition or expectant)
S1	(DE "PREGNANT women" OR DE "PREGNANCY" OR DE "MOTHERS")

Scopus	TITLE-ABS-KEY (pregnan* OR childbearing OR "child bearing" OR gestation* OR placenta* OR parturition OR (expectant W/2 (mother OR wom?n*))) AND (TITLE-ABS-KEY (racist OR racism) OR (TITLE-ABS-KEY (prejudic* OR discrim* OR hostil* OR harass* OR oppress* OR bully* OR bias* OR injusti*) AND TITLE-ABS-KEY (raci* OR race* OR ethnic* OR culture* OR minorit*)))
Web of Science Core collection	TS= (pregnan* OR childbearing OR "child bearing" OR gestation* OR placenta* OR parturition OR (expectant near/2 (mother OR wom?n*))) AND (TS=(racist OR racism) OR (TS= (prejudic* OR discrim* OR hostil* OR harass* OR oppress* OR bully* OR bias* OR injusti*) AND TS= (raci* OR race* OR ethnic* OR culture* OR minorit*)))

Supplemental table 2. Overview adverse pregnancy outcomes included in the review

Neonatal adverse outcomes	Maternal adverse outcomes (any until six weeks postpartum)
• Fetal or perinatal death (miscarriage or stillbirth)<br>• Preterm birth (<37 weeks)<br>• Gestational age at birth; infant body size at birth (weight, length, and head circumference); & small and large-for-gestational-age (defined as a birthweight below the 10th percentile or above 90th percentile for gestation according to fetal sex on standardized birth-weight charts, respectively)<br>• Admission to NICU or neonatal nursery<br>• Toleration of birthing process (5 minute Apgar score)<br>• Fetal growth restriction	• Post-partum haemorrhage<br>• Death<br>• Stroke<br>• Placental abruption<br>• Preterm labour rupture of the membranes<br>• Pulmonary embolism requiring therapy, Deep-vein thrombosis<br>• Pregnancy-induced hypertension (gestational Hypertension), Eclampsia, Preeclampsia<br>• Gestational diabetes

Supplemental table 3. Grading Quality of Evidence and Strength of Recommendations (GRADE)

Outcomes	No. participants (studies)	Quality of evidence (GRADE)	Risk of bias	Imprecision	Inconsistency	Indirectness	Publication Bias
Preterm birth	40824 (18 studies)	Very Low	-1	0	0	0	-1
Low birthweight	8704 (12 studies)	Very Low	-1	-1	0	0	n/a
Small-for-gestational-age	13226 (4 studies)	Very Low	-1	-1	-1	0	n/a
Hypertensive disorders of pregnancy	9470 (1 study)	Low	0	0	n/a	0	n/a

van Daalen KR, *et al. BMJ Global Health* 2022; 7:e009227. doi: 10.1136/bmjgh-2022-009227

Supplemental table 4. Preferred Items for Systematic Reviews and Meta-analyses (PRISMA) Checklist.[30]

Section and Topic	Item #	Checklist item	Location where item is reported
**TITLE**			
Title	1	Identify the report as a systematic review.	Page 1
**ABSTRACT**			
Abstract	2	See the PRISMA 2020 for Abstracts checklist.	Page 2
**INTRODUCTION**			
Rationale	3	Describe the rationale for the review in the context of existing knowledge.	Page 3-4
Objectives	4	Provide an explicit statement of the objective(s) or question(s) the review addresses.	Page 3-4
**METHODS**			
Eligibility criteria	5	Specify the inclusion and exclusion criteria for the review and how studies were grouped for the syntheses.	Page 6
Information sources	6	Specify all databases, registers, websites, organisations, reference lists and other sources searched or consulted to identify studies. Specify the date when each source was last searched or consulted.	Page 5-6
Search strategy	7	Present the full search strategies for all databases, registers and websites, including any filters and limits used.	Supplement Table 1
Selection process	8	Specify the methods used to decide whether a study met the inclusion criteria of the review, including how many reviewers screened each record and each report retrieved, whether they worked independently, and if applicable, details of automation tools used in the process.	Page 5-9
Data collection process	9	Specify the methods used to collect data from reports, including how many reviewers collected data from each report, whether they worked independently, any processes for obtaining or confirming data from study investigators, and if applicable, details of automation tools used in the process.	Page 5-9
Data items	10a	List and define all outcomes for which data were sought. Specify whether all results that were compatible with each outcome domain in each study were sought (e.g. for all measures, time points, analyses), and if not, the methods used to decide which results to collect.	Supplement Table 2
	10b	List and define all other variables for which data were sought (e.g. participant and intervention characteristics, funding sources). Describe any assumptions made about any missing or unclear information.	Page 7
Study risk of bias assessment	11	Specify the methods used to assess risk of bias in the included studies, including details of the tool(s) used, how many reviewers assessed each study and whether they worked independently, and if applicable, details of automation tools used in the process.	Page 7
Effect measures	12	Specify for each outcome the effect measure(s) (e.g. risk ratio, mean difference) used in the synthesis or presentation of results.	Page 7
Synthesis methods	13a	Describe the processes used to decide which studies were eligible for each synthesis (e.g. tabulating the study intervention characteristics and comparing against the planned groups for each synthesis (item #5)).	Page 5-6
	13b	Describe any methods required to prepare the data for presentation or synthesis, such as handling of missing summary statistics, or data conversions.	Page 7-8
	13c	Describe any methods used to tabulate or visually display results of individual studies and syntheses.	Page 7-8
	13d	Describe any methods used to synthesize results and provide a rationale for the choice(s). If meta-analysis was performed, describe the model(s), method(s) to identify the presence and extent of statistical heterogeneity, and software package(s) used.	Page 7-8

van Daalen KR, *et al. BMJ Global Health* 2022; 7:e009227. doi: 10.1136/bmjgh-2022-009227

	13e	Describe any methods used to explore possible causes of heterogeneity among study results (e.g. subgroup analysis, meta-regression).	Page 7-8
	13f	Describe any sensitivity analyses conducted to assess robustness of the synthesized results.	Page 7-8
Reporting bias assessment	14	Describe any methods used to assess risk of bias due to missing results in a synthesis (arising from reporting biases).	Page 8
Certainty assessment	15	Describe any methods used to assess certainty (or confidence) in the body of evidence for an outcome.	Page 7
**RESULTS**			
Study selection	16a	Describe the results of the search and selection process, from the number of records identified in the search to the number of studies included in the review, ideally using a flow diagram.	Page 9, Figure 1
	16b	Cite studies that might appear to meet the inclusion criteria, but which were excluded, and explain why they were excluded.	NA
Study characteristics	17	Cite each included study and present its characteristics.	Page 9
Risk of bias in studies	18	Present assessments of risk of bias for each included study.	Page 9-10
Results of individual studies	19	For all outcomes, present, for each study: (a) summary statistics for each group (where appropriate) and (b) an effect estimate and its precision (e.g. confidence/credible interval), ideally using structured tables or plots.	Table 2
Results of syntheses	20a	For each synthesis, briefly summarise the characteristics and risk of bias among contributing studies.	Page 9-13
	20b	Present results of all statistical syntheses conducted. If meta-analysis was done, present for each the summary estimate and its precision (e.g. confidence/credible interval) and measures of statistical heterogeneity. If comparing groups, describe the direction of the effect.	Page 11-13
	20c	Present results of all investigations of possible causes of heterogeneity among study results.	Page 11-13
	20d	Present results of all sensitivity analyses conducted to assess the robustness of the synthesized results.	Page 11-12, Supplement Figures 1-5
Reporting biases	21	Present assessments of risk of bias due to missing results (arising from reporting biases) for each synthesis assessed.	Supplement figure 6
Certainty of evidence	22	Present assessments of certainty (or confidence) in the body of evidence for each outcome assessed.	Supplement tables 3-6
**DISCUSSION**			
Discussion	23a	Provide a general interpretation of the results in the context of other evidence.	Page 14
	23b	Discuss any limitations of the evidence included in the review.	Page 15-16
	23c	Discuss any limitations of the review processes used.	Page 15-16
	23d	Discuss implications of the results for practice, policy, and future research.	Page 16-17
**OTHER INFORMATION**			
Registration and	24a	Provide registration information for the review, including register name and registration number, or state that the review was not registered.	Page 5

protocol	24b	Indicate where the review protocol can be accessed, or state that a protocol was not prepared.	Page 5
	24c	Describe and explain any amendments to information provided at registration or in the protocol.	NA
Support	25	Describe sources of financial or non-financial support for the review, and the role of the funders or sponsors in the review.	Page 18
Competing interests	26	Declare any competing interests of review authors.	Page 18
Availability of data, code and other materials	27	Report which of the following are publicly available and where they can be found: template data collection forms; data extracted from included studies; data used for all analyses; analytic code; any other materials used in the review.	Page 9
